# Accessing Long-Lived, Highly Stable Phosphine-Ligand-Free Palladium Hydrides via Palladium–Micelle Synergy

**DOI:** 10.1021/jacs.6c02702

**Published:** 2026-04-20

**Authors:** Karanjeet Kaur, Tharique N. Ansari, Ramesh Hiralal Choudhary, Manisha Bihani, Maarten Nachtegaal, Adam H. Clark, Jacek B. Jasinski, Fabrice Gallou, Sachin Handa

**Affiliations:** † Department of Chemistry, 2628 University of Missouri, Columbia, Missouri 65211, United States; ‡ Department of Chemistry, 429572 University of Louisville, Louisville, Kentucky 40292, United States; § Materials Characterization, Conn Center for Renewable Energy Research, 5170 University of Louisville, Louisville, Kentucky 40292, United States; ∥ Center for Photon Science, Villigen CH-5232, Switzerland; ⊥ Center for Energy and Environmental Sciences PSI, Villigen CH-5232, Switzerland; # Department of Chemistry, ETH Zürich, Zürich CH-8093, Switzerland; ∇ Chemical and Analytical Development, 111826 Novartis Pharma AG, Basel 4056, Switzerland

## Abstract

Palladium hydrides (PdH_*x*_) are significant to hydrogen-transfer chemistry, yet their prolonged aqueous and air instability as well as their reliance on strong ligands for stabilization have limited their practical applications. Herein, we report dynamic micelle-enabled stable PdH_*x*_ nanoparticles that are readily generated and suspended within nonionic micelles. This synthesis process employs Pd­(OAc)_2_ as the Pd precursor, MeMgBr as the reductant, amphiphile PS-750-M as shielding nonionic micelles, and water as the dispersion medium. These hydride-rich nanophases persist for more than 1 year in air-saturated moisture, overcoming the canonical fragility of such metal hydrides in protic media. The micellar architecture provides hydrophobic compartments that shield PdH_*x*_ from proton-induced decomposition while maintaining access to H_2_ and substrate for catalysis. These hydrides were found to be basic in nature, as evidenced by external base-free catalytic detriflation, a transformation otherwise known to generate triflic acid in situ. Pd K-edge X-ray absorption and ^1^H nuclear magnetic resonance spectroscopy confirm the presence of persistent PdH_*x*_ species. The long-term stability and catalytic activity of these PdH_*x*_@micelles are significant for materials chemistry and catalysis.

## Graphical abstract

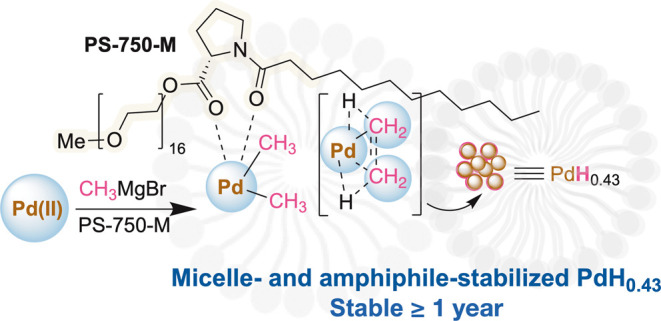

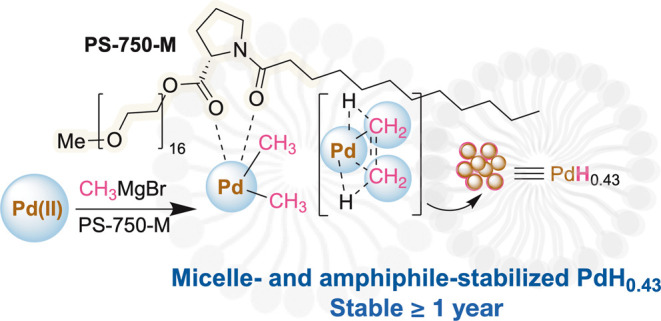

## Introduction

Palladium hydrides (PdH_*x*_) occupy a distinctive position in the field of catalysis,^1,2^ hydrogen storage,^3–5^ and electrochemical energy conversion.^6,7^ Hydrogen absorption onto Pd nanoparticles yields interstitial hydrides that modulate electronic structure,^3,8^ Pd–Pd distances,^9,10^ surface chemistry,^11^ and thereby catalytic selectivity.^12,13^ Of the two PdH_*x*_ structures reported in the literature, α-PdH_*x*_ has a lower concentration of hydride than β. Accordingly, β-PdH_*x*_, due to its abundance, can be used in organometallic catalysis relevant to organic synthesis.^2,13^ However, such isolated hydrides are known to be unstable in water.^2,14,15^ Literature precedents highlight the dynamics of α- and β-phase hydrides, particularly their formation and desorption in nanocrystals, where facets, vertices, and particle size significantly affect hydrogen uptake and release.^14–16^ Shape-controlled β-PdH_0.43_ nanocrystals have been synthesized in *N,N*-dimethylformamide (DMF). In this study, the role of DMF as a solvent, hydride source, and potentially as a capping agent formed in situ by its decomposition to CO and *N*,*N*-dimethylamine ligands was not studied.^17^ These β-PdH_0.43_ have been shown to retain activity for methanol oxidation, enabling studies in electrocatalysis and demonstrating the facet-dependent behavior of (100) versus (111) surfaces. Other established PdH_*x*_ synthesis protocols rely on organic media, capping ligands, or gaseous hydrogen/CO.^18–21^ Nonetheless, despite such precedents, to the best of our knowledge, there is no general, scalable approach to generate phosphine-ligand-free PdH_*x*_ nanoparticles that remain stable under moisture and air at ambient conditions for up to one year.

Despite the applications of PdH_*x*_ in hydrogen-transfer catalysis,^12^ hydrogen storage,^3–5^ and electrochemical energy conversion,^6,7^ their practical use is constrained by their rapid decomposition in water and air,^15,16^ necessitating strictly anhydrous, oxygen-free conditions. Access to PdH_*x*_ species that are both phosphine-ligand-free and stable in aqueous, aerobic media would enable hydride-transfer chemistry under truly mild practical conditions, thereby expanding their utility in organic synthesis, hydrogen-shuttling processes, and electrocatalysis. Stable PdH_*x*_ nanophases could provide long-lived, regenerable hydrides for transformations such as hydrofunctionalizations^13^ or base-free reductions while also enabling mechanistic interrogation of hydride insertion, diffusion, and phase behavior under liquid-phase conditions directly relevant to advanced chemistry. By overcoming the long-standing incompatibility between PdH_*x*_ and aqueous environments, such materials would open a new platform for aqueous-phase hydride catalysis.

Given the significance and scarcity of PdH_*x*_, which remains stable in both water and air for extended periods and can be prepared in water, we propose the development of highly stable PdH_*x*_ species in an aqueous environment by harnessing the compartmentalization effect of aqueous micelles. The operating principle for the synthesis of such PdH_*x*_ lies in the metal-micelle cooperativity that shields the surface of in situ-generated nanoparticles of PdH_*x*_. The micelles of PS-750-M are known to act as a new platform for running water-sensitive reactions in water,^22–24^ combining compartmentalization, increased effective molarity, and protective microenvironments for transient species.^24–27^ This supports our approach to extending shielding and stabilizing hydride-bearing, phosphine-ligand-free surfactant-encapsulated nanopalladium within nonionic micelles, thereby achieving water stability without the use of conventional ligands. In addition to impacting processing, strong ligands often impede the mass transfer of substrates to the surface in aqueous systems and block active sites, leading to reduced catalytic activity.^28,29^ We hypothesize that PS-750-M micelles stabilize nanoparticles of PdH_*x*_ through three synergistic effects: (1) reversible carbonyl anchoring and weak chelation at the micellar interface or interior, damping surface reconstruction and suppressing hydride desorption; (2) hydrophobic compartmentalization that minimizes water attack but preserves H_2_ access during reactions; and (3) facet/vertex selectivity and strain management controlled by micelles via slow nucleation, reducing α ↔ β oscillations that likely drive H loss in bulk liquids. Additionally, the micelle-stabilized PdH_*x*_ can be characterized spectroscopically. An increase in Pd–Pd bond length derived from extended X-ray absorption fine structure (EXAFS) spectra as well as characteristic changes in the X-ray absorption near edge structure (XANES) spectral features point to the presence of interstitial hydride,^30–33^ while proton nuclear magnetic resonance (^1^H NMR) signals associated with metal hydrides indicate the presence of hydrides at or near the nanoparticle surface.

## Results and Discussion

Following our working hypothesis, we designed a synthesis strategy involving transmetalation of the alkyl group onto the Pd precursor, generating a pseudostable Pd species. This intermediate can undergo either α-hydride elimination or alkyl–alkyl metathesis, ultimately leading to the formation of PdH_*x*_ species and a volatile byproduct that rapidly escapes from the reaction mixture (Scheme 1). Based on these considerations, methyl can be the ideal alkyl group due to its small size and the formation of ethylene gas as the metathesis byproduct, which readily evolves from the reaction medium. This process facilitates the generation of PdH_*x*_ species. Treatment of Pd­(OAc)_2_ with 2 equiv of MeMgBr in THF, followed by gentle heating in an aqueous 5 wt % PS-750-M solution, afforded PdH_*x*_ nanoparticles. The resulting PdH_*x*_ can be isolated by filtration or evaporation and stored in the medium or as a solid under ambient air and moist conditions without significant decomposition for up to one year (vide infra). This synthetic protocol required only ca. 45 min (for details, see Supporting Information, Page S2).

**Scheme 1:**
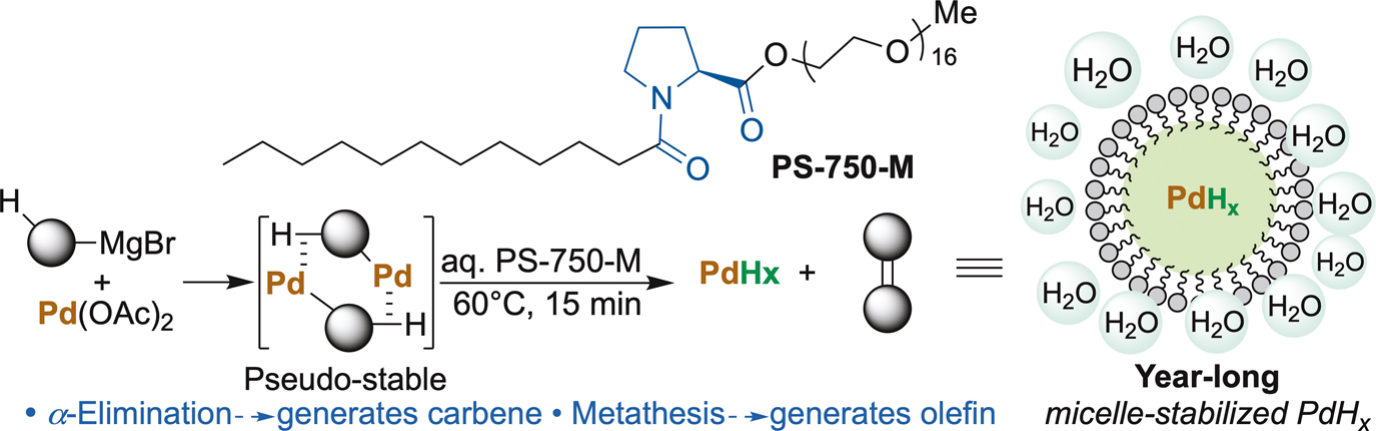
A General Approach to the Synthesis of Water-Stable PdH_*x*_ in Water

High-resolution transmission electron microscopy (HRTEM) (Figure 1A–C; for details, see Supporting Information, Pages S3, S4) and high-angle annular dark-field scanning transmission electron microscopy (HAADF-STEM) (Figure 1D) of the resulting dried solid revealed the morphology and nanoparticle nature of the PdH_*x*_-containing material. The particles were found to be somewhat spherical, surrounded by PS-750-M molecules. The average size of the nanoparticles was 4.8 ± 0.7 nm (see Supporting Information, Figure S4, Page S4). From the images, it appears that the nanoparticles were likely interconnected with the aid of PS-750-M.

**Figure 1:**
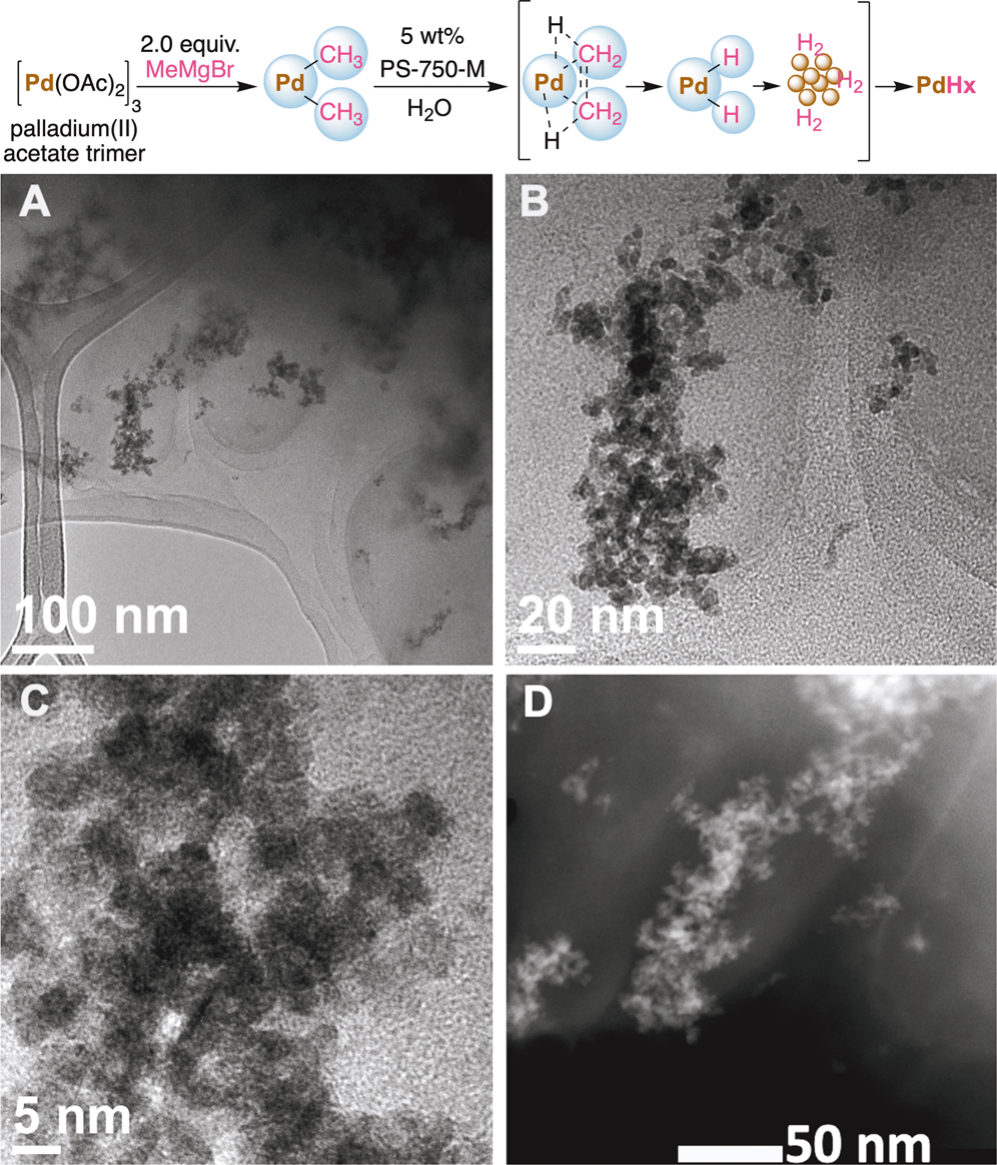
Synthesis scheme of micellar nanoparticles of PdH*_x_* and imaging of the dried nanoparticles. (A–C) High-resolution transmission electron microscopy images. (D) High-angle annular dark-field scanning transmission electron microscopy images.

EXAFS and XANES analyses of a one-year-old sample stored under air at ambient temperature confirmed the presence of hydride species (designated as MB2), revealing mixed Pd(0)/PdH_*x*_ states. The loss of hydride through reductive elimination may have resulted in the formation of Pd(0), which then deposits on the surface of the remaining PdH_*x*_. Nevertheless, PdH_*x*_ formation was validated through Pd K-edge XAS measurements (Figure 2A). EXAFS fitting of the Fourier-transformed EXAFS spectra (Figure 2B,C) yielded a Pd–Pd coordination number of 8.8 and an average bond distance of 2.81 Å, representing an expanded lattice in PdH_*x*_, similar to a face-centered cubic (fcc) Pd cluster (Table 1). Hydride stoichiometry was quantified from EXAFS fitting and by using the following empirical relation:^31^

$$\Delta R\left(T\right)/R_{0}\left(T\right)=\left(0.0666\cdot x\right)-\left(0.0164x^{2}\right)$$

where Δ*R*(*T*) represents the change in Pd–Pd bond distance within Pd nanoparticles upon hydrogen insertion, *R*_0_(T) is the bond distance in hydrogen-free Pd nanoparticles, and *x* denotes the H/Pd ratio. This equation is valid for 0 < *x* < 0.5 and relies on the accurate determination of bond distances from EXAFS data. From the measurements of the MB2 sample, *x* = 0.43, corresponding to a composition of PdH_0.43_.

**Figure 2:**
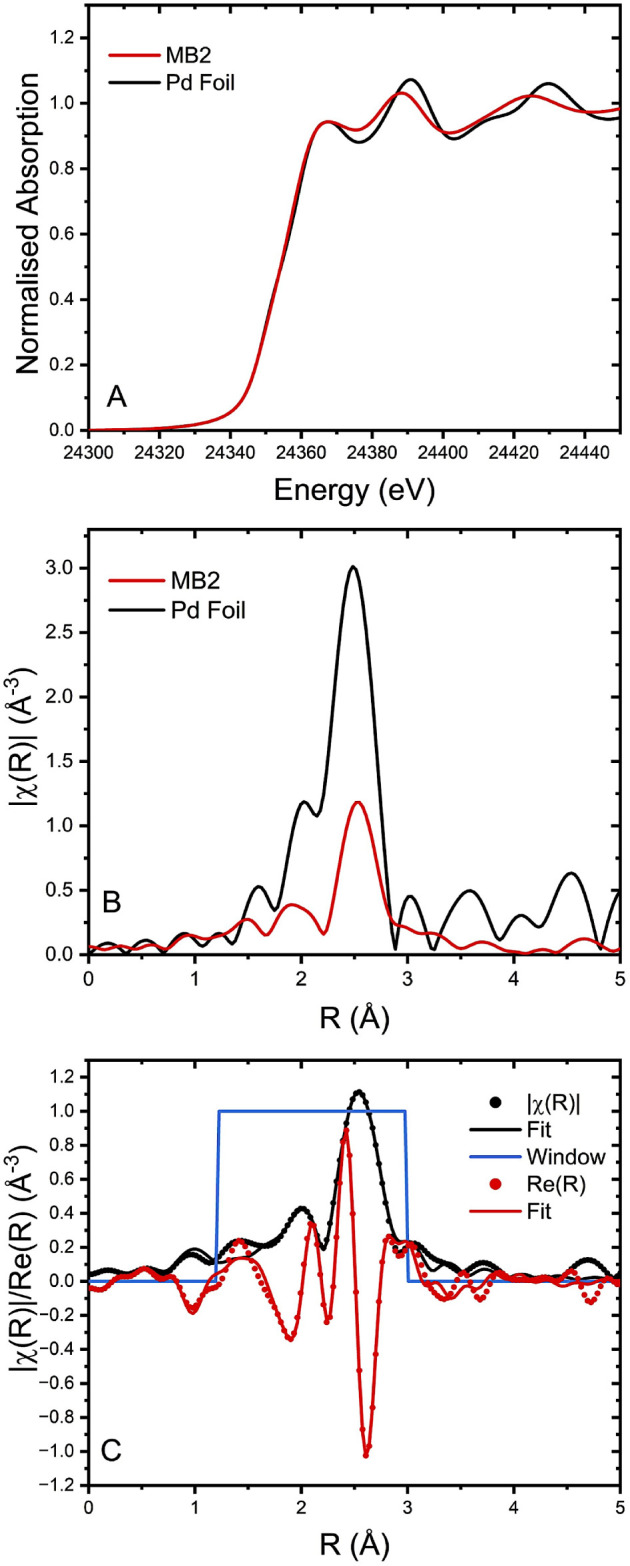
X-ray absorption spectra of Pd and PdH*_x_*. (A) Pd XANES and (B, C) Pd EXAFS fitting to the FT EXAFS spectra.

**Table 1:** Best Fit Parameters to the Pd K Edge EXAFS of MB2^a^

Scattering Path	*N*	σ^2^ (Å^2^)	*R* (Å)
Pd–Pd	8.8 ± 0.8	0.010 ± 0.001	2.81 ± 0.01

a *N* is the number of nearest neighbors, *R* is the Pd–Pd bond distance, and σ^2^ is the pseudo Debye–Waller factor.

Based on XAS analysis, the stability of these PdH_*x*_ nanophases in water contradicts the prevailing view that metal hydrides are inherently short-lived in moisture. We attribute this longevity to the synergistic effects of the PS-750-M matrix, including microphase partitioning, surface coating of PdH_*x*_ by PS-750-M, and micelle encapsulation, which sequesters the nanoparticles from the continuous aqueous phase, dramatically reducing proton-induced hydride quenching.

Proton nuclear magnetic resonance (^1^H NMR) spectroscopy was utilized to support the hypothesis regarding the mechanism of formation and stabilization, as well as to confirm the presence of PdH_*x*_ species (for details, see Supporting Information, Pages S6–S8). Upon addition of MeMgBr to the Pd precursor suspension, transmetalation occurred, transferring methyl groups from MeMgBr to Pd, as depicted in Scheme 1 and Figure 1. This was evidenced by a distinct CH_3_ resonance at −0.93 (Figure 3A, at 0 h), corresponding to Pd­(CH_3_)_2_, which differs from the MeMgBr signal, typically appearing at −1.95 to −1.54 ppm.^34^ Simultaneously, PdH_*x*_ signals appeared at −0.70 and −0.72 ppm,^18,35^ suggesting that ethylene formation likely proceeds via either α-elimination or a metathesis pathway. This inference is supported by the detection of ethylene at 5.38 ppm in ^1^H NMR (for details, see Supporting Information, Figure S6, Page S7), a byproduct consistent with the α-elimination process. The signal at −0.17 ppm is likely from the presence of less stable α-PdH_*x*_. Over the course of 5 h, the Pd­(CH_3_)_2_ signal completely disappeared, while the PdH_*x*_ signal intensified and persisted, indicating conversion to a hydride-rich species. During this period, the resonance at −0.72 ppm coalesces with the signal at −0.70 ppm, which likely reflects the migration of hydride species from the NP surface into the interior. In addition to the compartmentalization effect, this inward hydride redistribution may play a key role in stabilizing PdH_*x*_ in aqueous media, as it allows surfactant molecules to occupy the majority of surface coordination sites, while the hydrides reside in protected internal positions shielded from water. After 30 h, a loss of intensity of PdH_*x*_ was also observed, possibly due to either a reduction in *d*_6_-DMSO or changes in micellar compartmentalization caused by DMSO required for the NMR analysis. Notably, PdH_*x*_ decomposed immediately in the absence of PS-750-M, as demonstrated by the ^1^H NMR study (for details, see Supporting Information, Page S9).

**Figure 3:**
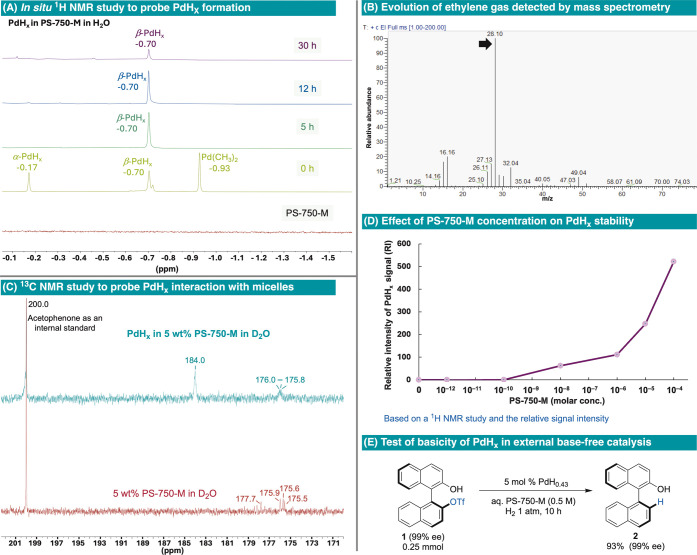
Experiments supporting the mechanism of formation of PdH_0.43_. (A) *In-situ* ^1^H NMR analysis of the reaction mixture. (B) Gas chromatography–mass spectrometry supports the evolution of ethylene from the metathesis reaction. (C) PdH*_x_*-micelle interaction study. (D) The relationship between the stability of PdH*_x_* and the concentration of PS-750-M. (E) Test to confirm the basicity of PdH_0.43_ in external base-free catalysis.

Furthermore, the formation of ethylene via α-elimination or a metathesis pathway was also supported by gas chromatography–mass spectrometry (GC–MS), which showed a mass of 28.1 Da at a retention time of 0.68 min for ethylene (Figure 3B; to see more details and images with retention times, see Supporting Information, Figure S19b, Pages S18, S19). The ethylene signal differed from that of atmospheric nitrogen, although both have similar masses (retention time of 0.51 min for N_2_; for details, see Supporting Information, Pages S18, S19). Together with XAS, NMR, and GC–MS analyses, the observations confirm the chemical identity of the PdH_0.43_ material and provide mechanistic insights into its formation.

The interaction between PdH_0.43_ and PS-750-M micelles, which likely contributed to the extended stability of PdH_0.43_, was investigated using ^13^C NMR spectroscopy (Figure 3C; for details, see Supporting Information, Page S17). The amidic and ester carbonyl resonances of PS-750-M were monitored. In a 5 wt % PS-750-M solution in D_2_O, carbonyl signals appeared in the range of 175–178 ppm, which likely reflects the presence of dynamic micelles of varying sizes and morphologies. Upon the introduction of PdH_0.43_, a pronounced downfield shift was observed, with an intense signal at 184 ppm. This shift suggests potential binding of PdH_0.43_ with PS-750-M in either a monomeric or micellar environment. Additionally, residual or PdH_0.43_-free micelles of PS-750-M were evident from signals at 175.8–176 ppm. The dynamic behavior of micelles and the exchange between PdH_0.43_-bound and PdH_0.43_-free micelles likely caused signal broadening at 184 ppm. This behavior distinguishes the binding modes of micelles from those of traditional ligands and capping agents, which are responsible for keeping the catalytically active sites available (vide infra).^24^ These observations confirm that PdH_0.43_ interacts with PS-750-M, as supported by the ^13^C NMR analysis.

The presence of PS-750-M micelles is crucial for stabilizing PdH_*x*_, as PdH_*x*_ decomposes immediately in their absence (Figure 3D; see Supporting Information, Page S9). Our data further confirm that the pronounced stabilization of PdH_*x*_ arises from a micellar effect rather than from simple surface coating by the surfactant PS-750-M. To substantiate this conclusion, we prepared PdH_*x*_ at six different concentrations of PS-750-M—below the critical micelle concentration (CMC), near the CMC, and above the CMC—and systematically monitored hydride stability over 0, 12, and 24 h. Plotting the relative ^1^H hydride signal intensity as a function of surfactant concentration shows that substantial stabilization occurs at or above 10^–6^ M, consistent with a true micellar stabilization mechanism, although some degree of stabilization can still occur through the surface binding of monomers (Figure 3D; for further details, see Supporting Information, Pages S9–S14). Additional control experiments further supported this claim; when PdH_*x*_ was generated in *t*-BuOH using PS-750-M present solely in its monomeric (nonmicellar) form, only short-lived stabilization was observed (see Figure S16 and Pages S14, S15 in Supporting Information). A pronounced decrease in the PdH_*x*_ hydride signal occurred within 4 h, and most of the hydride species had decomposed by 8 h, consistent with a weak and transient surface-coating effect. These results clearly show that long-term aqueous stabilization of PdH_*x*_ relies more on micellar encapsulation than on simple surfactant adsorption, although the latter can still provide some stability; the effect of micelles is more pronounced.

The basic character of PdH_0.43_ was further validated through an external base-free detriflation reaction with the catalytic use of PdH_0.43_, a transformation typically performed in the presence of a stoichiometric base, a conventional Pd catalyst, and a hydrogen atmosphere. In this study, the monotriflate of (*S*)-BINOL **1** (with 99% ee) was subjected to hydrogenation under a hydrogen atmosphere using 5 mol % of PdH_0.43_ suspended in an aqueous solution of PS-750-M, affording product **2** in 93% isolated yield (Figure 3E; for details, see Supporting Information, Pages S22–S26). This catalytic activity arises from the use and regeneration of PdH_0.43_ under a hydrogen atmosphere. This outcome supports the basic nature of PdH_0.43_—these hydride species are not only stable but also regenerative while preserving their inherent basicity.

## Conclusions

In conclusion, our study bridges the synthesis, stability, and characterization of phosphine-ligand-free micelle-encapsulated PdH_0.43_ in water, revealing metal–micelle cooperativity that extends beyond Pd(0)/Pd­(II) nanoparticles to solid-solution hydrides. It reveals the hydride-stabilizing function of nonionic micelles in aqueous conditions. By demonstrating the long-term storage of hydride in micellar nanoreactors and a catalytic scope under mild conditions, this work establishes PdH_0.43_@PS-750-M micelles as a potential platform for liquid-phase hydride chemistry.

## Supplementary Material



## Notes

General information, materials synthesis, detailed analytical data, details of catalytic activity tests, and GC–MS and NMR data (PDF)
