# Supplementary material for: Accessing Long-Lived, Highly Stable Phosphine-Ligand-Free Palladium Hydrides via Palladium–Micelle Synergy
Source: J Am Chem Soc. 2026 Apr 20;148(17):18204–9. doi: 10.1021/jacs.6c02702 (PMC13143402; doi:10.1021/jacs.6c02702)
Supplement: Supplementary file 1 [file ja6c02702_si_001.pdf]

Accessing Long-Lived, Highly Stable Phosphine-Ligand-Free Palladium Hydrides via Palladium–Micelle Synergy

Karanjeet Kaur,[‖†#] Tharique N. Ansari,[†#] Ramesh Hiralal Choudhary,[‖#] Manisha Bihani,[†#] Maarten Nachtegaal,[⊥∇^] Adam H. Clark,[⊥] Jacek B. Jasinski,[§] Fabrice Gallou,[‡] Sachin Handa[†‖*]

[‖]Department of Chemistry, University of Missouri, Columbia, MO 65211, United States
[†]Department of Chemistry and §Materials Characterization, Conn Center for Renewable Energy Research, University of Louisville, Louisville, KY 40292, United States
[⊥]Center for Photon Science and [∇]Center for Energy and Environmental Sciences PSI, Villigen, CH-5232, Switzerland
[^]Department of Chemistry, ETH Zürich, CH-8093, Zürich, Switzerland
[‡]Chemical and Analytical Development, Novartis Pharma AG, Basel 4056, Switzerland
[#]These authors equally contributed.

*Email: sachinhanda@missouri.edu

Supporting Information

Contents **Page #**

1. General experimental details S1

2. Synthesis of palladium hydride S2

3. HRTEM, EDS analysis, and particle size distribution of palladium hydride S3, S4

4. XAS of palladium hydride (MB2) S5

5. NMR experiments S6-S17

6. GC-MS experiments S18, S19

7. Control experiments S20, S21

8. Basic nature of $PdH_{0.43}$: Catalytic activity S22-S29

9. References S30

1. GENERAL EXPERIMENTAL DETAILS

All manipulations were carried out under an inert atmosphere unless otherwise noted. TLC plates (UV 254 indicator, aluminum-backed, 175-225 µm thickness) and silica gel (standard grade, 230 – 400 mesh) were purchased from Silicycle. $Pd(OAc)_2$ and MeMgBr (3M in Et_2O or THF) were purchased from Sigma-Aldrich. Ethyl acetate, tetrahydrofuran, and hexanes were purchased from Fisher Scientific. Aqueous solution of surfactant (PS-750-M) was prepared using distilled water and thoroughly purged with argon before use. Deuterated NMR solvents were purchased from Cambridge Isotopes Laboratories or Sigma-Aldrich. The reactions were performed in a 10 mL reaction vial unless otherwise mentioned. All NMR spectra were recorded at 25 °C on Varian Unity INOVA (400, 500 MHz) and on a Bruker AVANCE III HD spectrometer (400, 500, and 600 MHz) with a commercial 5 mm probe. GC-MS data were obtained using a Thermo Scientific Mass Spectrometer. Reported chemical shifts are referenced to residual solvent peaks.[1]

2. SYNTHESIS OF PALLADIUM HYDRIDE

This sample was named MB2.

Procedure. Oven-dried microwave vial (4.0 mL) containing a PTFE-coated magnetic stir bar was charged with anhydrous $Pd(OAc)_2$ (100 mg, 0.445 mmol). The reaction vial was closed with a Suba-Seal septum. The reaction vial was gently evacuated and backfilled with argon. This cycle was repeated three additional times. Dry THF (0.2 mL) was added, and the mixture was stirred for 10 minutes at room temperature (Figure S1, a). Then, MeMgBr (0.45 mL, 1.33 mmol, 3.0 M solution in THF or Et_2O) was added at the same temperature, and the mixture was stirred at 45 ^{o}C for the next 15 minutes. At this point, the formation of a dark brownish-black waxy solid was observed (Figure S1, b). The reaction mixture was allowed to cool to ambient temperature and quenched with 1 mL of aqueous solution of 5 wt% aqueous PS-750-M. The mixture was stirred for an additional 15 minutes at rt. Afterwards, the liquids were removed under vacuum to obtain a black, aqueous solid mixture (Figure S, 1c). The remaining mixture was dried overnight under vacuum to yield 112 mg of shiny black PdH_X particles (Figure S1, d).

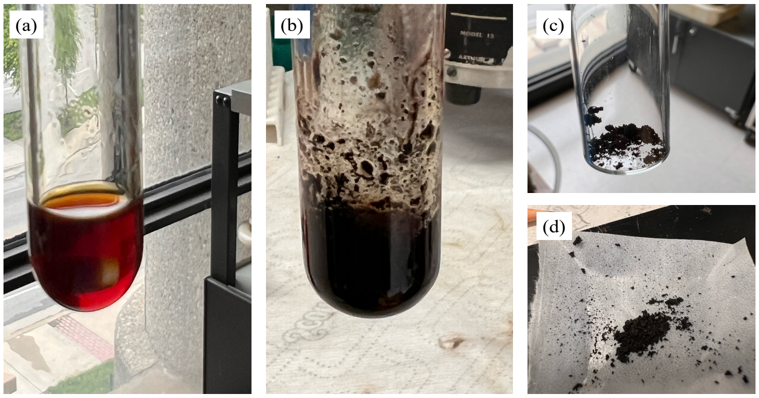

Figure S1. (a) $Pd(OAc)_2$ in THF, (b) After addition of MeMgBr and PS-750-M, (c) After removal of liquids under vacuum, stored in a vial, (d) Free-flowing dry palladium hydride particles on a weighing paper.

3. HRTEM, EDS ANALYSIS, AND PARTICLE SIZE DISTRIBUTION OF PALLADIUM HYDRIDE

The analysis was conducted using a 200-kV field-emission gun FEI Tecnai F20 microscope and specimens prepared by drop-casting catalyst dispersions onto commercial copper grid-supported amorphous holey carbon films.

The sample prepared for HRTEM was further analyzed using element mapping to confirm the presence of palladium by energy-dispersive X-ray spectroscopy (EDS) (Figure S2). The higher % of magnesium than palladium indicated that more magnesium resides at the surface of the nanoparticle.

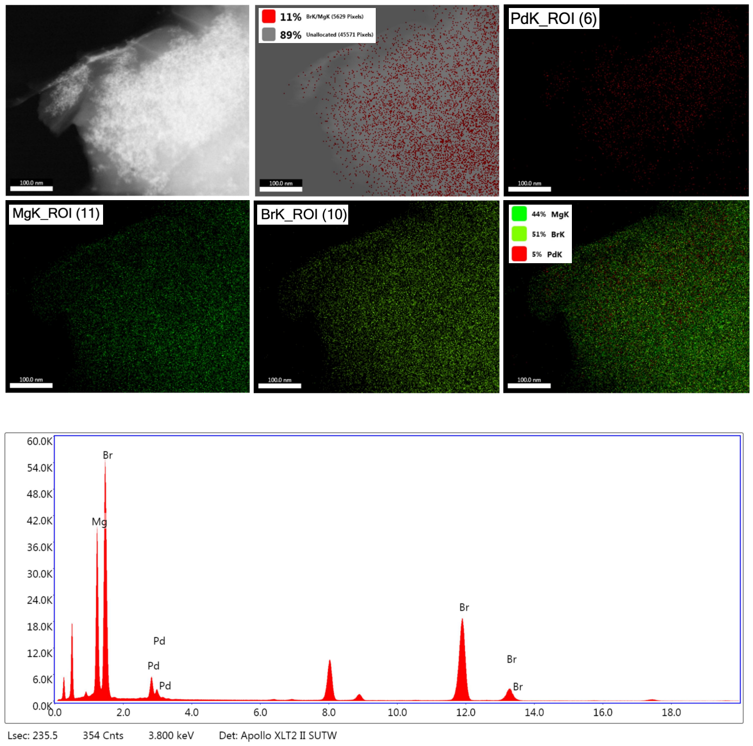

Figure S2. Elemental mapping and distribution in EDS analysis.

Table S1. eZAF Smart Quant Results

Element	Weight %	Atomic %	Net Int.	Error %
MgK	90.29	96.87	1240.50	1.89
BrK	9.25	3.02	1772.10	10.43
PdK	0.46	0.11	64.50	17.19

Particle size distribution

By random selection, the following images were used for calculating particle size distribution:

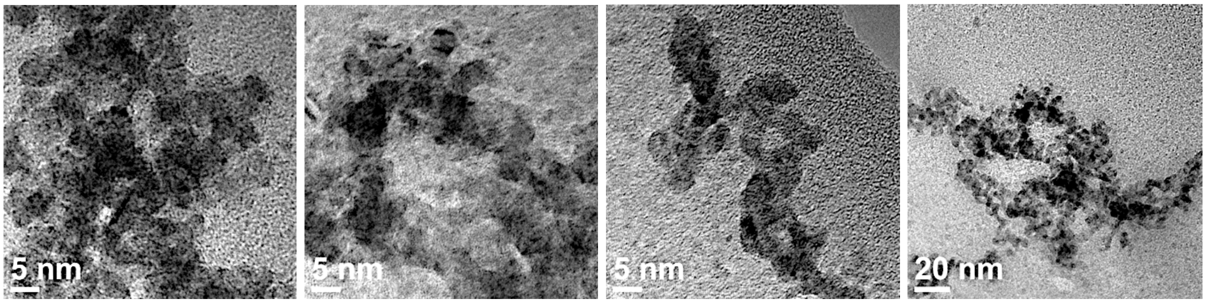

Figure S3. Randomly selected images for determining the average particle size.

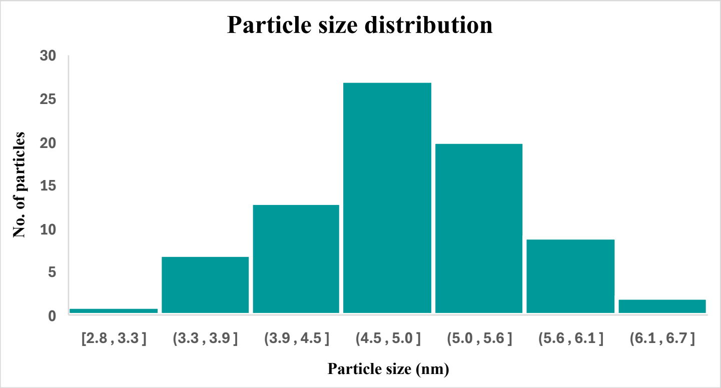

Figure S4. Particle size distribution.

The average particle size = 4.84 nm.

4. XAS OF PALLADIUM HYDRIDE (MB2)

The sample was prepared following the procedure in Section 2, page S2. The characterization was performed at the Paul Scherrer Institute. The ex-situ Pd K edge measurements were performed at the SuperXAS beamline of the SLS. The X-ray beam subtended from the 2.9 T superbend magnet was collimated with a Pt coated mirror at 2.64 mrad and subsequently monochromatized with a water cooled channel cut Si 111 quick scanning monochromator. The beam was further focused using a Pt coated toroidal double focussing mirror yielding a beam size of approximately 1000 x 100 um (H x V). Gas filled ionisation chambers were used to measure the incident beam intensity with the sample measured in fluorescence yield with a PIPS detector. Simultaneously a Pd reference foil was measured for energy calibration and determination of the amplitude reduction factor for the EXAFS analysis.

5. NMR EXPERIMENTS

5.1. In-situ ^{1}H NMR analysis of reaction mixture

Procedure. To a clean and oven-dried NMR tube, $Pd(OAc)_2$ (9 mg, 0.04 mmol) was added. The tube was closed with a rubber septum. The tube was gently evacuated and backfilled with nitrogen. This was repeated three additional times. Then, 0.4 mL DMSO-d_6 was added, and the mixture was vortexed to dissolve the catalyst. MeMgBr (40 µL, 0.12 mmol, 3 M in Et_2O) was slowly transferred via micro-liter syringe, and after gentle vortexing, 0.1 mL of 3 wt% PS-750-M dissolved in d_6-DMSO was added. The resulting mixture was analyzed by ^{1}H NMR at different time intervals: t (h) = 0, 5, 12, and 30 (Figure S5). DMSO was added to obtain a highly soluble sample, while PS-750-M was needed to stabilize the Pd-H for detection.

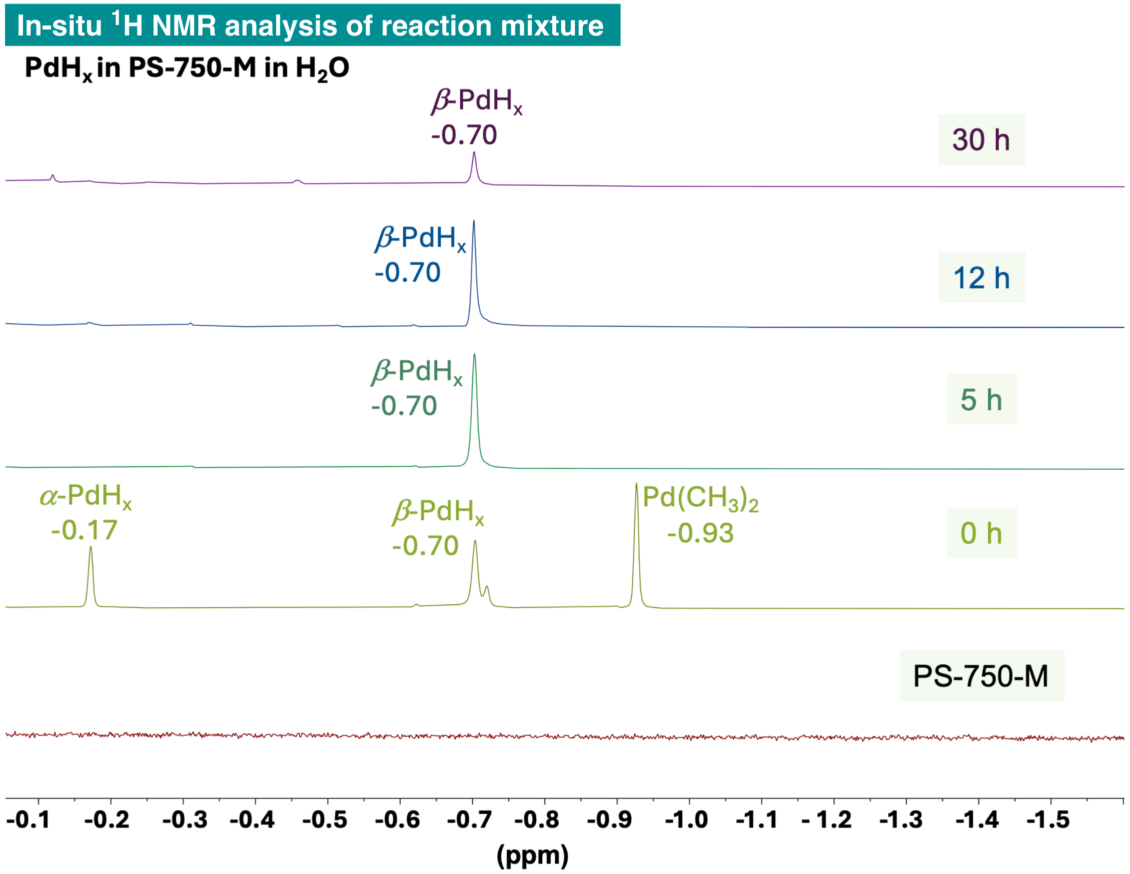

Figure S5. In-situ ^{1}H NMR analysis of reaction mixture.

5.2. To detect the evolution of ethylene gas during palladium hydride formation

The above sample was analyzed by ^{1}H NMR to monitor ethylene gas formation (Figure S6).

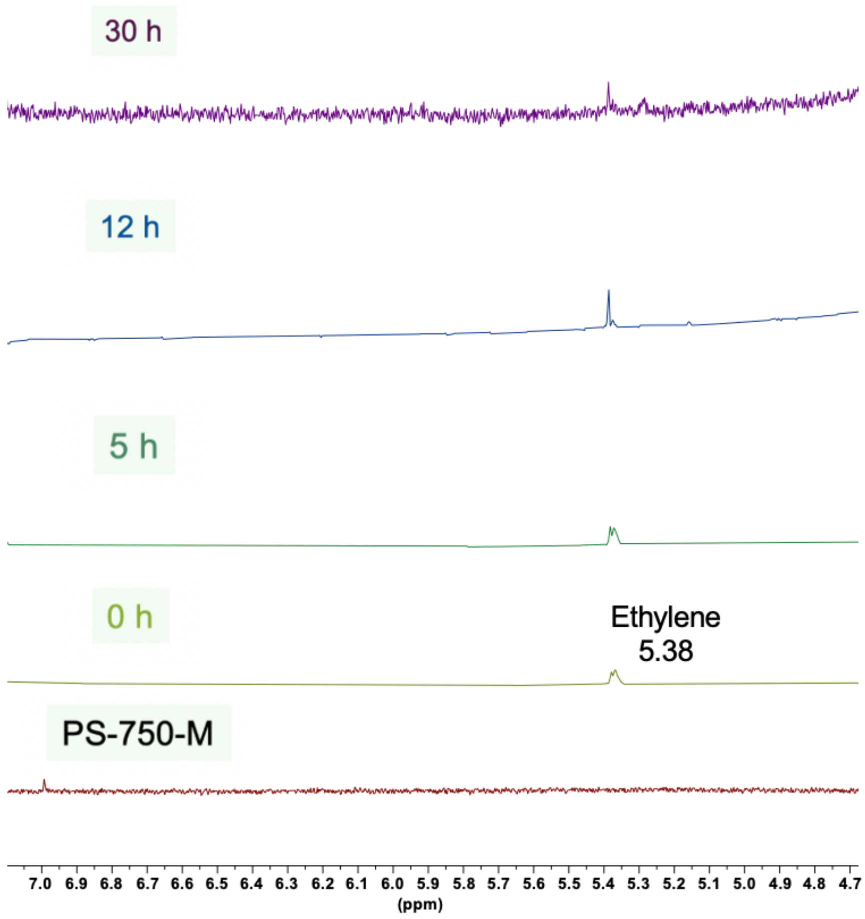

Figure S6. ^{1}H NMR indicating evolution of ethylene gas at 5.38 ppm.

5.3. ^{1}H NMR analysis for probing the amount of Grignard reagent required for the formation of PdH_x

The sample was prepared using the same procedure as described in Section 5.1, with 1.5 and 3 equivalents of Grignard reagent. The mixture was analyzed by ^{1}H NMR.

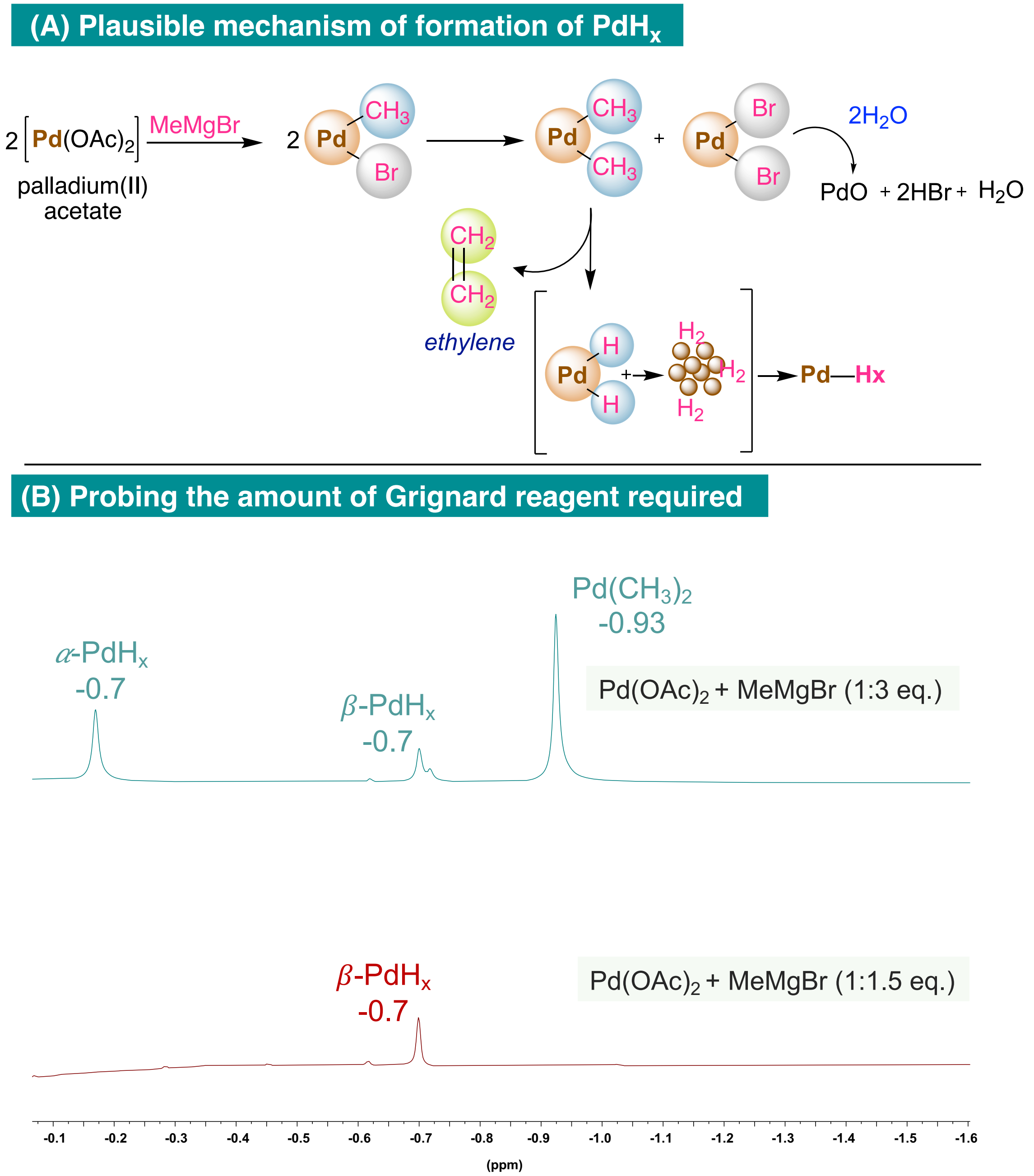

Figure S7. (A) Plausible mechanism of formation of PdH_x, (B)^{1}H NMR analysis for probing the amount of Grignard reagent required for the formation of PdH_x.

5.4. ^{1}H NMR analysis for accessing stability of PdH_x in neat water

Procedure. To a clean and oven-dried 4 mL reaction vial, $Pd(OAc)_2$ (9 mg, 0.04 mmol) was added. The vial was closed with a suba-seal rubber septum. The vial was gently evacuated and backfilled with nitrogen. This was repeated three additional times. Then, 0.4 mL d_6-DMSO was added, and the mixture was stirred vigorously at ambient temperature for 5 minutes to dissolve the solid content. MeMgBr (40 µL, 0.12 mmol, 3 M in Et_2O) was slowly transferred to the reaction vial via micro-liter syringe, and the mixture was stirred for an additional 2 minutes. The vial was opened, and the mixture was quickly transferred to an NMR tube sealed with a rubber septum under a nitrogen atmosphere with the aid of a syringe. The resulting mixture was analyzed by ^{1}H NMR to ensure PdH_x is formed, after which 0.1 mL freshly degassed distilled water was added, and the mixture was gently vortexed and reanalyzed by ^{1}H NMR (Figure S8). The signal from PdHx disappeared upon the addition of water.

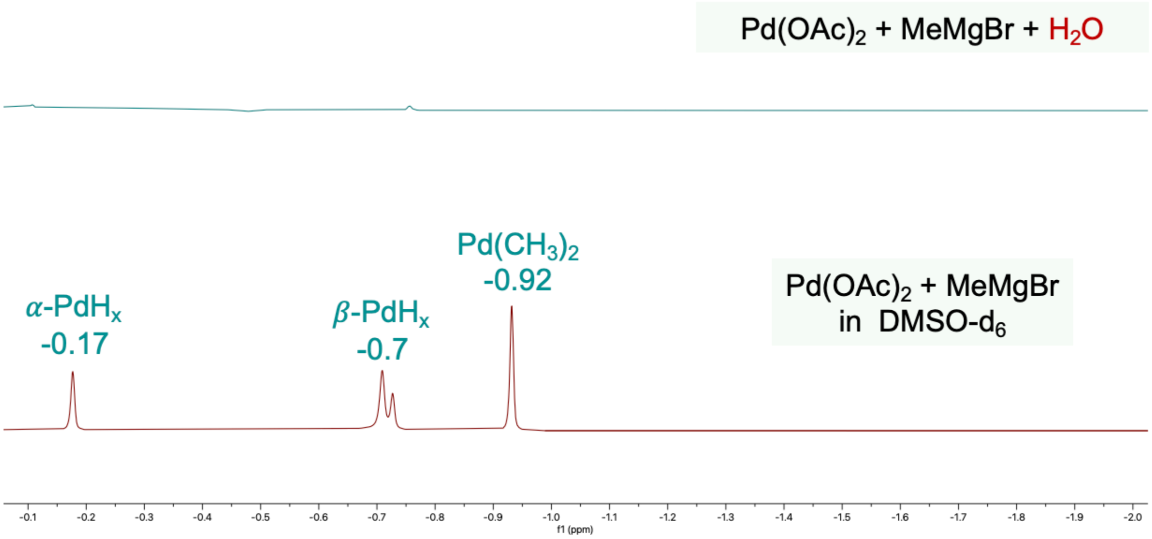

Figure S8. Stability of PdH_x in the absence of PS-750-M.

5.5. Correlation between PS-750-M concentration and relative intensity of PdH_x

Procedure. To a clean and oven-dried 4 mL reaction vial, $Pd(OAc)_2$ (9 mg, 0.04 mmol) was added. The vial was closed with a suba-seal rubber septum. The vial was gently evacuated and backfilled with nitrogen. This was repeated three additional times. Then, 0.4 mL d_6-DMSO was added, and the mixture was stirred vigorously at ambient temperature for 5 minutes to dissolve the solid contents. MeMgBr (40 µL, 0.12 mmol, 3 M in Et_2O) was slowly transferred to the reaction vial via micro-liter syringe, and the mixture was stirred for an additional 2 minutes. The vial was opened, and the mixture was quickly transferred to an NMR tube sealed with a rubber septum under a nitrogen atmosphere with the aid of a syringe. The resulting mixture was analyzed by ^1H NMR first to ensure that PdH_x is formed. Then, 0.1 mL of PS-750-M (conc. 10^{-12} M) was added, and after gently vortexing, the mixture was reanalyzed by ^1H NMR at different time intervals: t (h) = 0, 3, 5,12, and 24 (Figure S9).

The same procedure was followed for ^1H NMR analysis with PS-750-M (conc. 10^{-10} M, Figure S10) and (conc. 10^{-8}, 10^{-6}, 10^{-5}, 10^{-4} M; Figures S11, S12, S13, and S14).

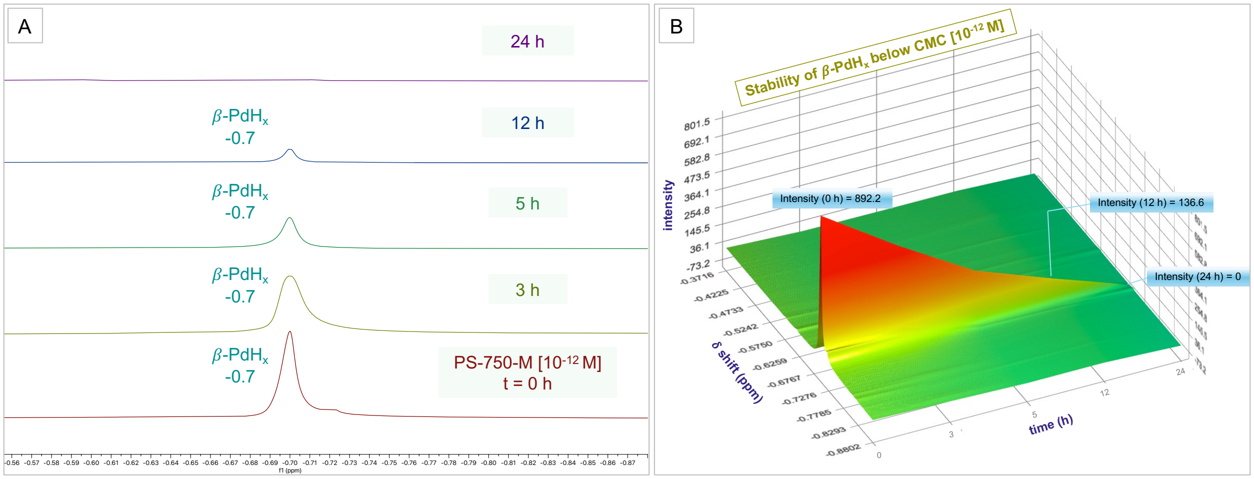

Figure S9. (A) ^1H NMR analysis of PdH_x using 10^{-12} M concentration of PS-750-M from 0 to 24 h, (B) 3D spectrum plot for PS-750-M (molar conc.) v/s relative intensity of PdH_x signal (RI) using 10^{-12} M concentration of PS-750-M at different time intervals.

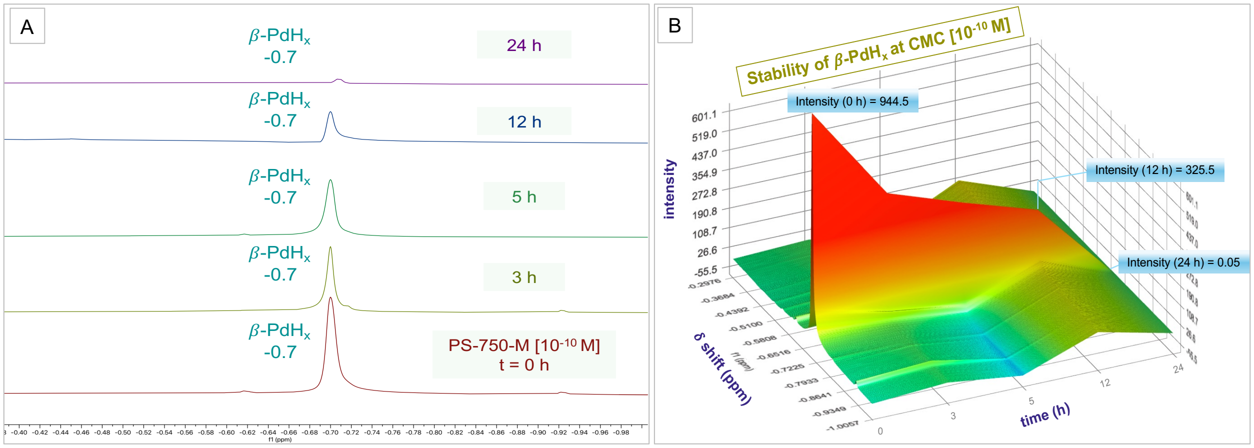

Figure S10. (A) ^{1}H NMR analysis of PdH_x using 10^{-10} M concentration of PS-750-M from 0 to 24 h, (B) 3D spectrum plot for PS-750-M (molar conc.) v/s relative intensity of PdH_x signal (RI) using 10^{-10} M concentration of PS-750-M at different time intervals.

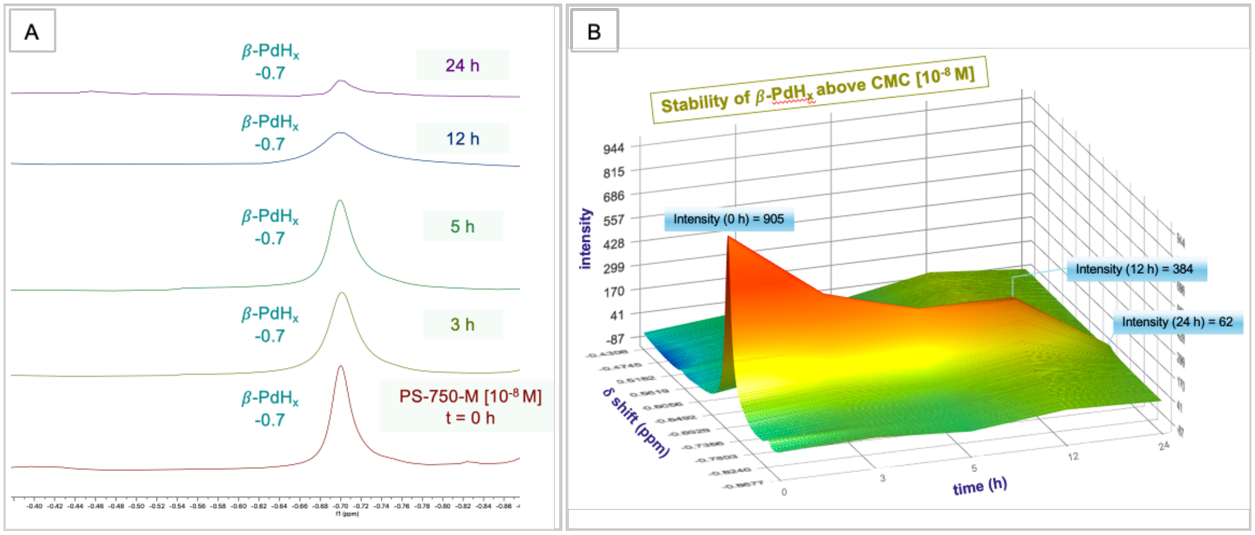

Figure S11. (A) ^{1}H NMR analysis of PdH_x using 10^{-8} M concentration of PS-750-M from 0 to 24 h, (B) 3D spectrum plot for PS-750-M (molar conc.) v/s relative intensity of PdH_x signal (RI) using 10^{-8} M concentration of PS-750-M at different time intervals.

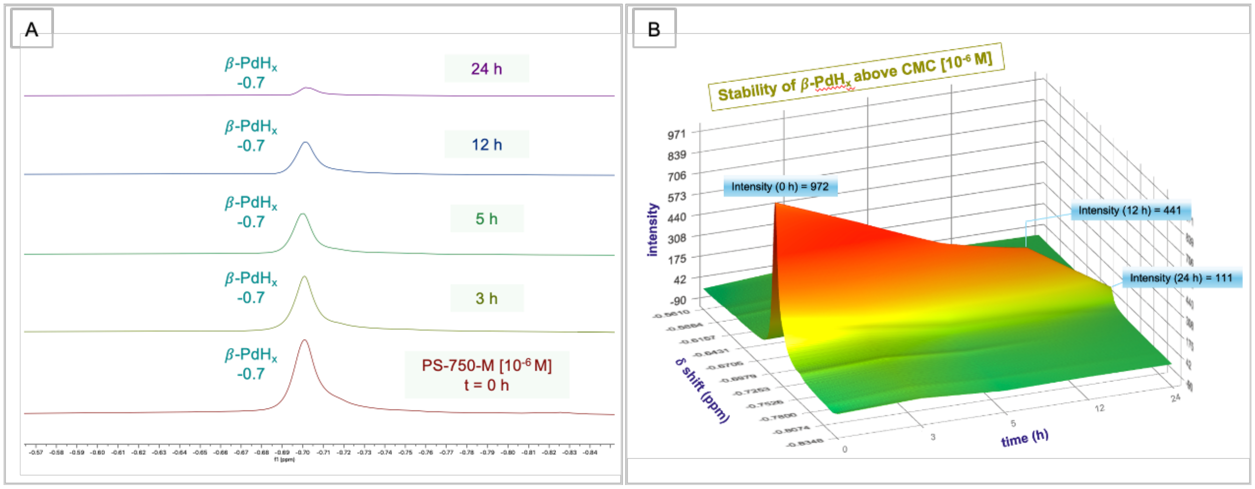

Figure S12. (A) ^{1}H NMR analysis of PdH_x using 10^{-6} M concentration of PS-750-M from 0 to 24 h, (B) 3D spectrum plot for PS-750-M (molar conc.) v/s relative intensity of PdH_x signal (RI) using 10^{-6} M concentration of PS-750-M at different time intervals.

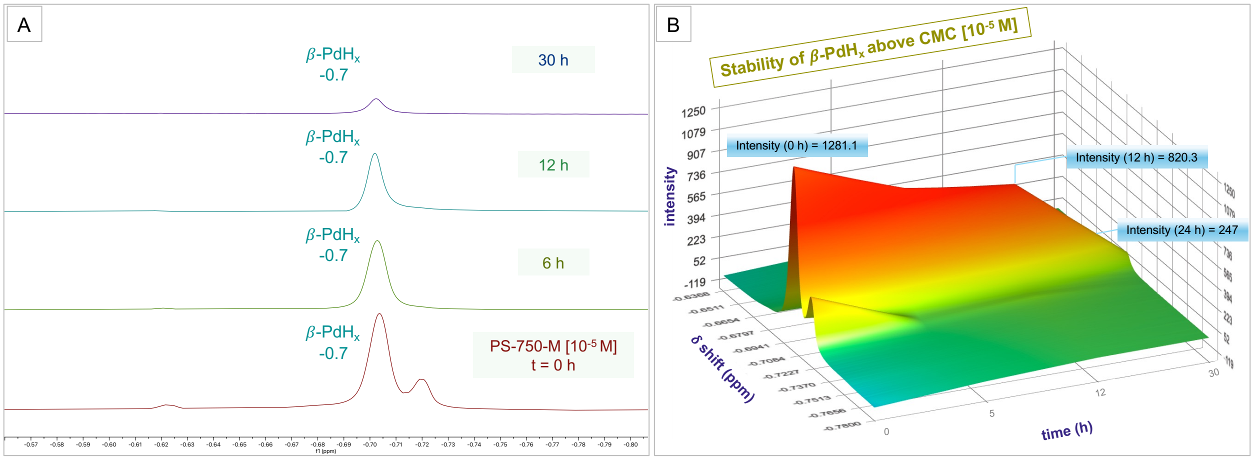

Figure S13. (A) ^{1}H NMR analysis of PdH_x using 10^{-5} M concentration of PS-750-M from 0 to 30 h, (B) 3D spectrum plot for PS-750-M (molar conc.) v/s relative intensity of PdH_x signal (RI) using 10^{-5} M concentration of PS-750-M at different time intervals.

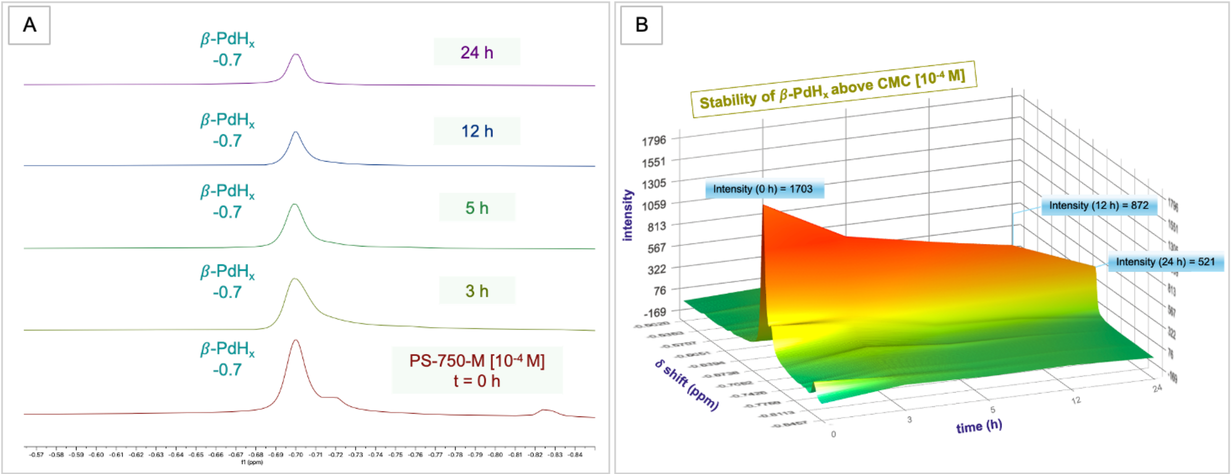

Figure S14. (A) ^{1}H NMR analysis of PdH_x using 10^{-4} M concentration of PS-750-M from 0 to 30 h, (B) 3D spectrum plot for PS-750-M (molar conc.) v/s relative intensity of PdH_x signal (RI) using 10^{-4} M concentration of PS-750-M at different time intervals.

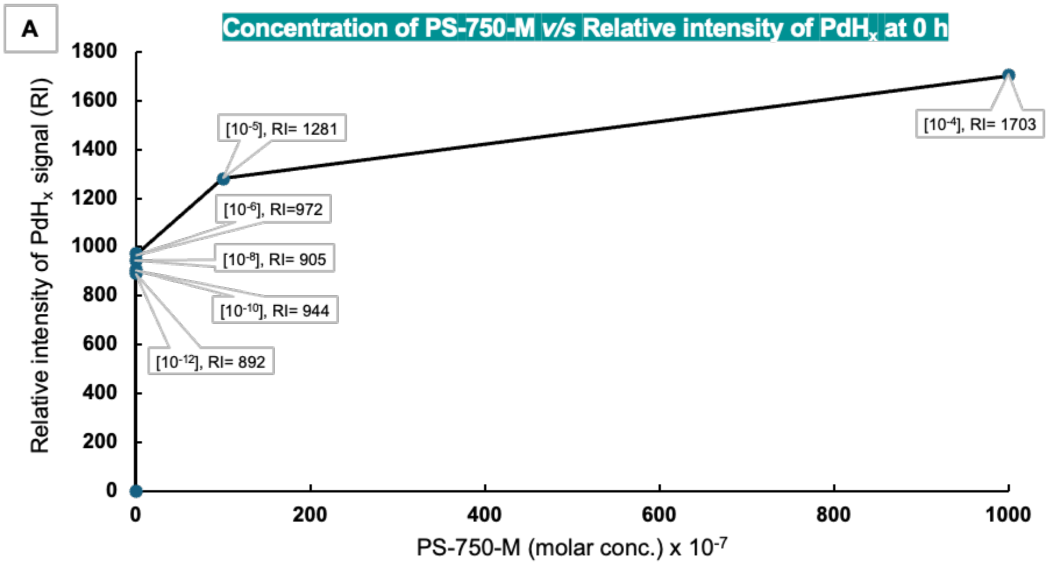

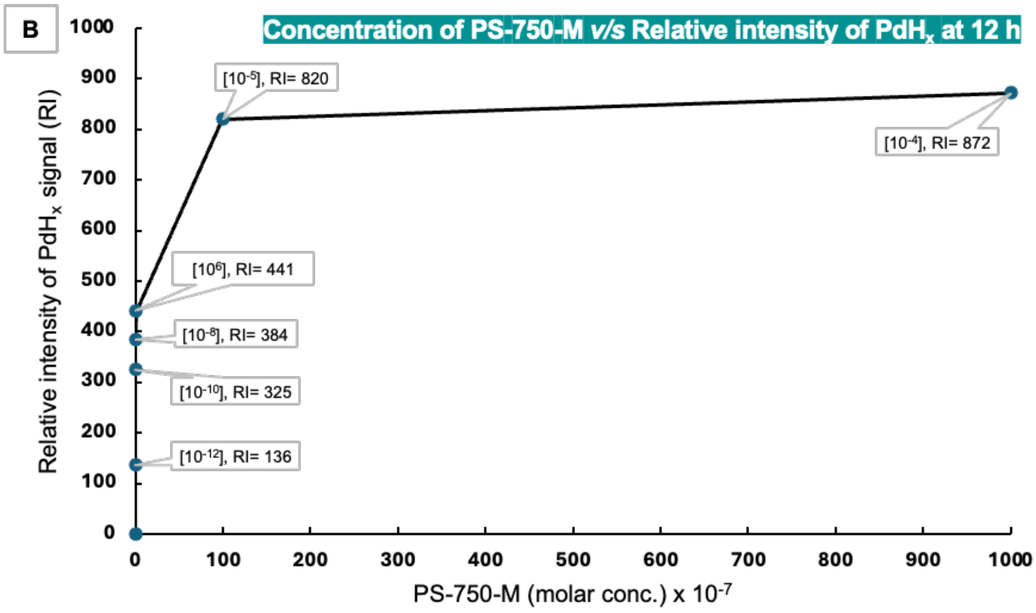

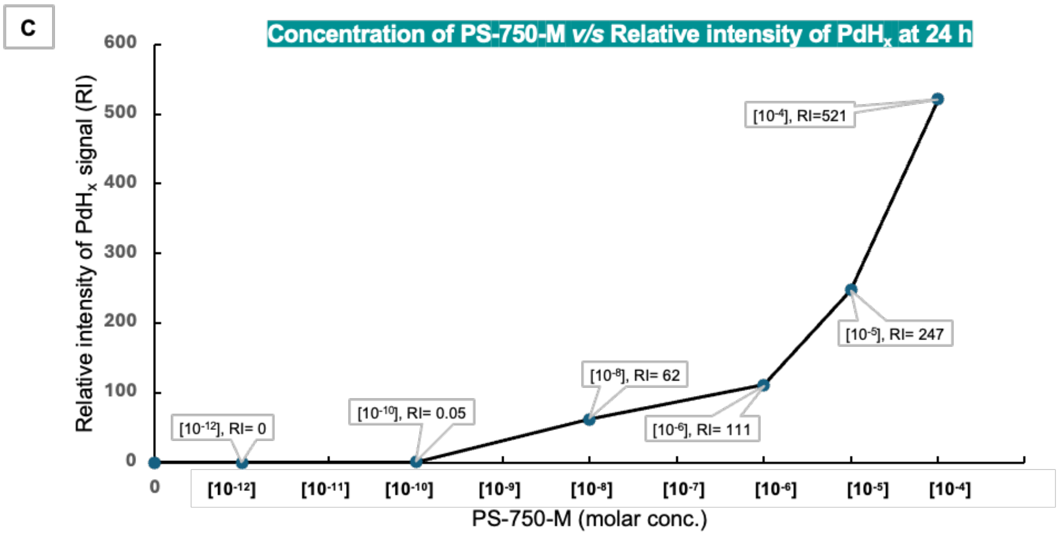

Figure S15. 2D spectrum for relative intensity of PdH_x signal *v/s* concentration of PS-750-M at (A) t = 0 h, (B) 12 h, and (C) 24 h.

5.6. ^{1}H NMR analysis for accessing stability of PdH_x using 3 wt% PS-750-M in t-BuOH

In t-BuOH, it is anticipated that PS-750-M will not form micelles, so the stabilization effect will arise from the coating of PdH_x by PS-750-M monomers.

Procedure. To a clean and oven-dried 4 mL reaction vial, $Pd(OAc)_2$ (9 mg, 0.04 mmol) was added. The vial was closed with a suba-seal rubber septum. The vial was gently evacuated and backfilled with nitrogen. This was repeated three additional times. Then, 0.4 mL DMSO-*d₆* was added, and the mixture was stirred vigorously at ambient temperature for 5 mins to dissolve the solid contents. MeMgBr (40 µL, 0.12 mmol, 3 M in Et_2O) was slowly transferred via micro-liter syringe, and the mixture was stirred for an additional 2 mins. The vial was opened, and the mixture was quickly transferred to an NMR tube sealed with a rubber septum under a nitrogen atmosphere with the aid of a syringe. The resulting mixture was analyzed by ^1H NMR to ensure the formation of PdH_x. Then, 0.1 mL of 3 wt% PS-750-M dissolved in anhydrous *t*-butanol was added, and after gently vortexing, the mixture was reanalyzed by ^1H NMR at different time intervals: t (h) = 0, 4, and 8. (Figure S16).

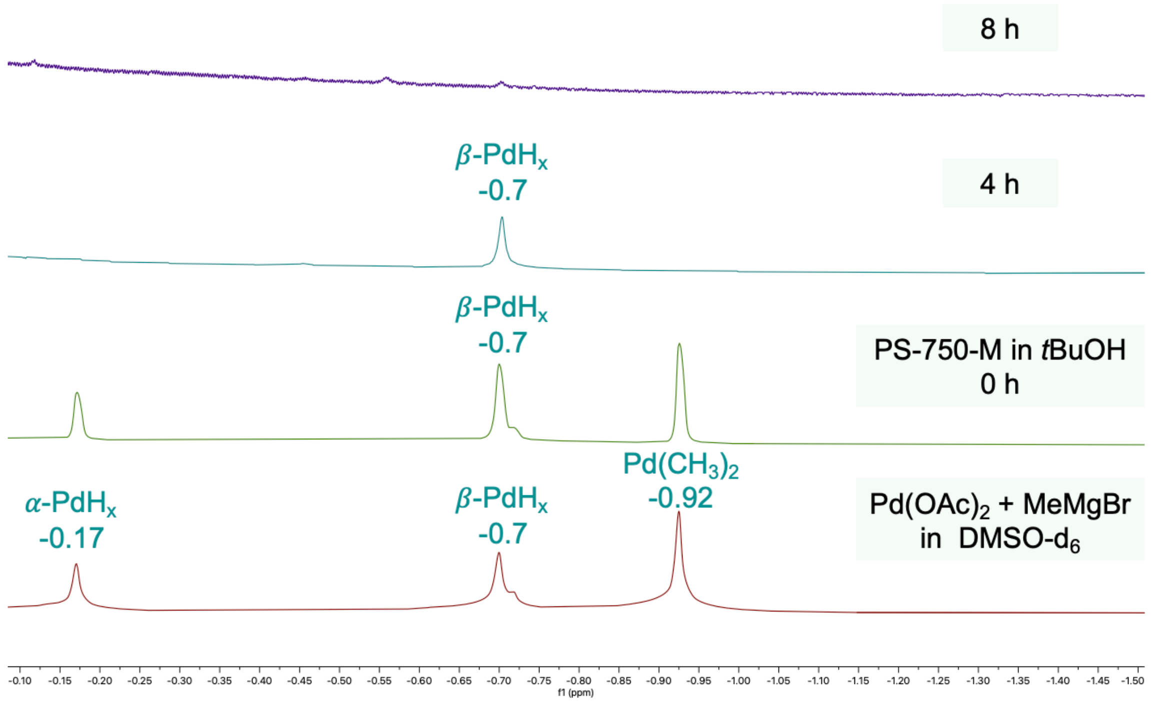

Figure S16. Stability of PdH_x with 3 wt. % PS-750-M in *t*Butanol.

5.7. ^{1}H NMR analysis for accessing the stability of PdH_x with 3 wt% sodium dodecyl sulfate (SDS)

Procedure. To a clean and oven-dried 4 mL reaction vial, $Pd(OAc)_2$ (9 mg, 0.04 mmol) was added. The vial was closed with a suba-seal rubber septum. The vial was gently evacuated and backfilled with nitrogen. This was repeated three additional times. Then, 0.4 mL DMSO-d_6 was added, and the mixture was stirred vigorously at ambient temperature for 5 mins to dissolve the solid contents. MeMgBr (40 µL, 0.12 mmol, 3 M in Et_2O) was slowly added to the mixture via micro-liter syringe, and the reaction mixture was stirred for an additional 2 mins. The vial was opened, and the mixture was quickly transferred to an NMR tube sealed with a rubber septum under a nitrogen atmosphere with the aid of a syringe. The resulting mixture was analyzed by ^{1}H NMR first to ensure the formation of PdH_x. Then, 0.1 mL of 3 wt% SDS was added. After gently vortexing the mixture was reanalyzed by ^{1}H NMR at different time intervals: t (h) = 0, 1, and 2. (Figure S17).

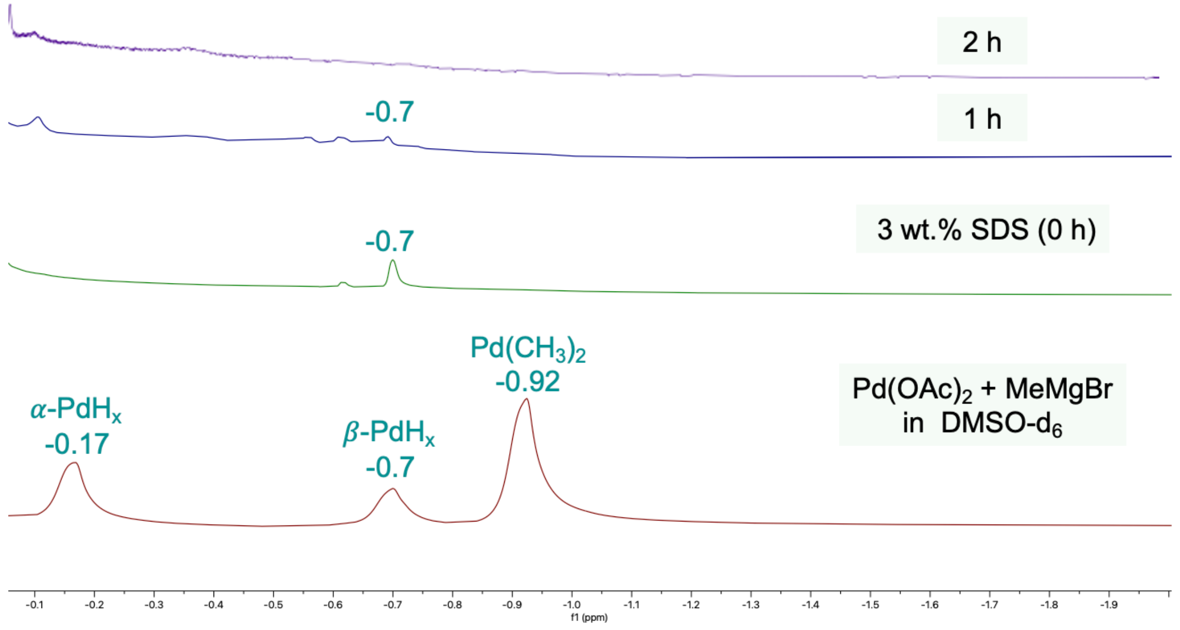

Figure S17. Stability of PdH_x with 3 wt. % SDS.

5.8. ^{13}C NMR study to understand the role of PS-750-M in the stabilization of in-situ synthesized PdH_x

Procedure. To a clean and oven-dried NMR tube, $Pd(OAc)_2$ (11.2 mg, 0.05 mmol) was added. The tube was closed with a rubber septum. The tube was gently evacuated and backfilled with nitrogen. This was repeated three additional times. Then, 0.2 mL dry THF was added, followed by slow addition of MeMgBr (50 µL, 0.05 mmol, 3M in Et_2O). The resulting mixture was vortexed for 2-3 minutes at room temperature. At this point, the mixture turned black, and the formation of nanoparticles was observed. Separately, 25 mg of PS-750-M was dissolved using 0.3 mL D_2O in a 2 mL vial. The vial was closed with a rubber septum and was maintained under positive nitrogen atmosphere. Then, the freshly prepared PS-750-M D_2O solution was introduced, and the mixture was vortexed for 5 minutes. The resulting mixture was analyzed by ^{13}C NMR (Figure S18). For comparison, a ^{13}C NMR spectrum was also obtained for the freshly prepared solution of PS-750-M in D_2O. Acetophenone, dissolved in DMSO, was suspended in the NMR tube as an internal standard within a sealed capillary. The ^{13}C NMR spectra for both samples were recorded at a frequency of 125 MHz.

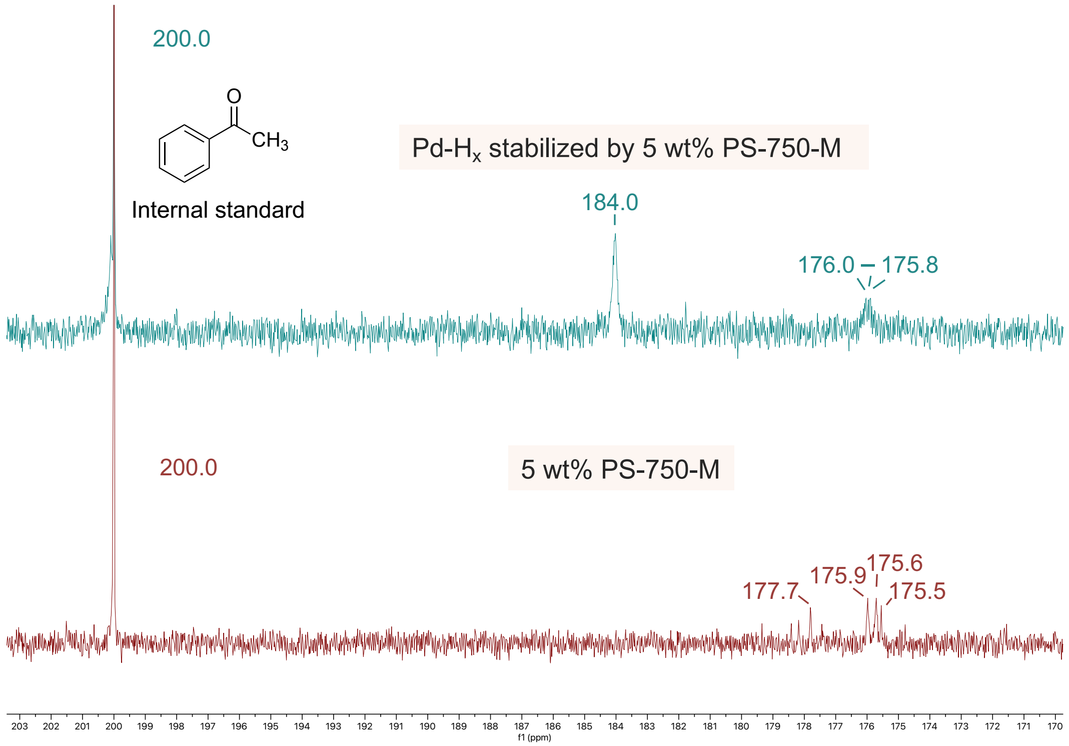

Figure S18. ^{13}C NMR analysis for stabilization of PdH_x with PS-750-M.

6. GC-MS EXPERIMENTS

6.1. To detect the evolution of ethylene gas

Sample preparation.

A) ***Ethylene as a standard***

Equipped a closely packed 4 mL vial with a balloon filled with ethylene gas, injected a gaseous sample into the GC-MS sample injector port, and looked for the mass of ethylene gas (Figure S19a).

B) ***The in situ release of ethylene gas during the palladium hydride formation in MB2.***

To an oven-dried vial (4.0 mL) containing a PTFE-coated magnetic stir bar was charged with $Pd(OAc)_2$ (50 mg, 0.22 mmol). The reaction vial was closed with a Suba-Seal septum. The reaction vial was gently evacuated and backfilled with argon. This cycle was repeated three additional times. Dry THF (0.2 mL) was added, and the mixture was stirred for ten minutes at room temperature. MeMgBr (0.15 mL, 0.45 mmol, 3.0 M solution in THF) was added; at this point, a sample was immediately drawn from the headspace of the vial with a microliter syringe and injected into the GC-MS sample injection port (Figure S19b).

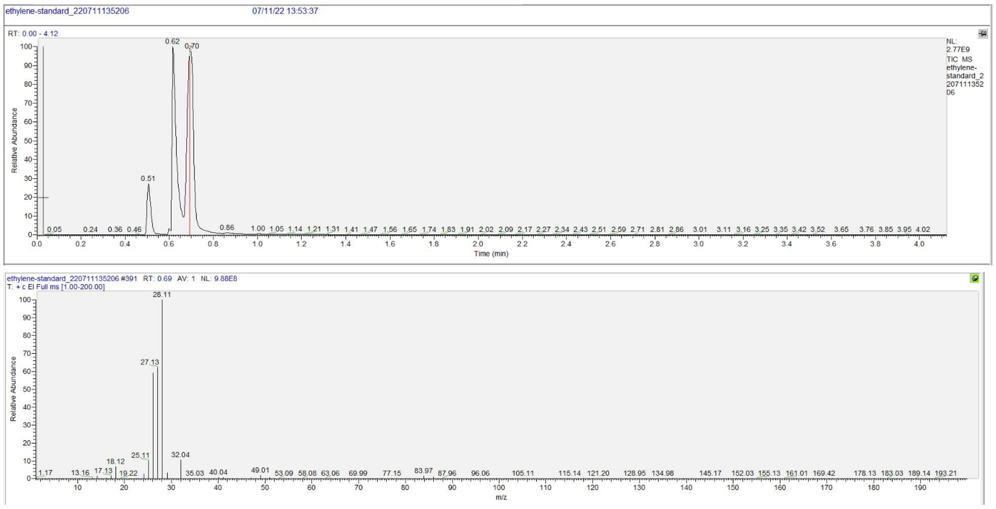

entry	retention time (minutes)	molecule
1	0.51	nitrogen
2	0.62	dichloromethane
3	0.7	ethylene gas

Figure S19a. GC-MS of standard ethylene gas.

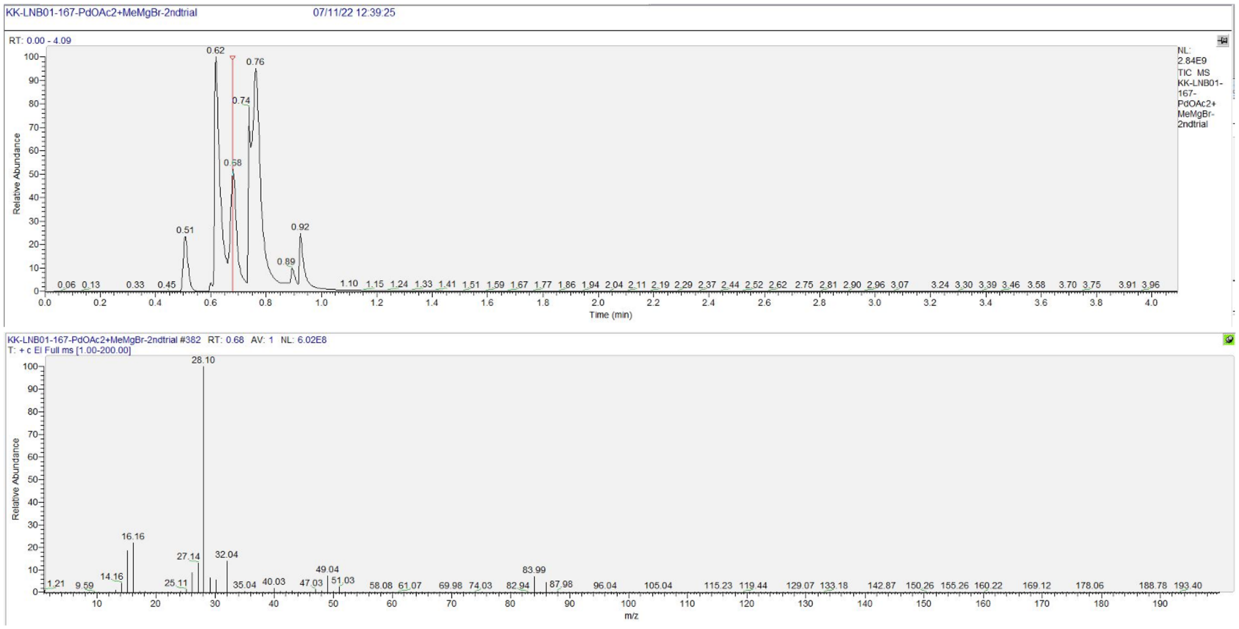

entry	retention time (minutes)	molecule
1	0.51	nitrogen
2	0.62	dichloromethane
3	0.68	ethylene gas
4	0.76	acetone
5	0.89	ethyl acetate
6	0.92	tetrahydrofuran

Figure S19b. GC-MS of evolved ethylene gas during the formation of palladium hydride.

Both neat ethylene and ethylene generated in situ from a reaction sample exhibited a signal at ca. 0.68 rt with m/z 28, confirming ethylene evolution during PdHx formation.

7. CONTROL EXPERIMENTS

7.1. Decomposition and regeneration of PdHx

Procedure. To an oven-dried vial (4.0 mL) containing a PTFE-coated magnetic stir bar, $Pd(OAc)_2$ (2.8 mg, 5 mol%) was charged. The reaction vial was closed with a subaseal septum. The reaction vial was gently evacuated and backfilled with nitrogen. This cycle was repeated twice more. Dry THF (0.5 mL) was added, and the mixture was stirred at 45 °C for 15 minutes. The mixture was allowed to cool to room temperature, and MeMgBr (9 µL, 0.025 mmol, 3.0 M solution in Et_2O) was added. Then the mixture was heated at 60 ºC for 1 h. Then, 0.8 mL freshly degassed 5 wt% aq. PS-750-M was introduced. The mixture was allowed to stir for the next 5 min at rt, followed by the addition of (*S*)-2'-hydroxy-[1,1'-binaphthalen]-2-yl trifluoromethanesulfonate) **1** (0.25 mmol), and the septum was wrapped with PTFE tape. A balloon filled with H_2 gas was introduced, and the mixture was stirred at 65 °C for 12 h (Scheme S1). After completion of the reaction time, the reaction vial was cooled to rt. The septum was removed, and 2 mL EtOAc was added to the reaction mixture to extract the product. The crude was analyzed by TLC using (7:3) hexanes/ethyl acetate as the TLC solvent system. No product was found on TLC, and all the starting material remained as is.

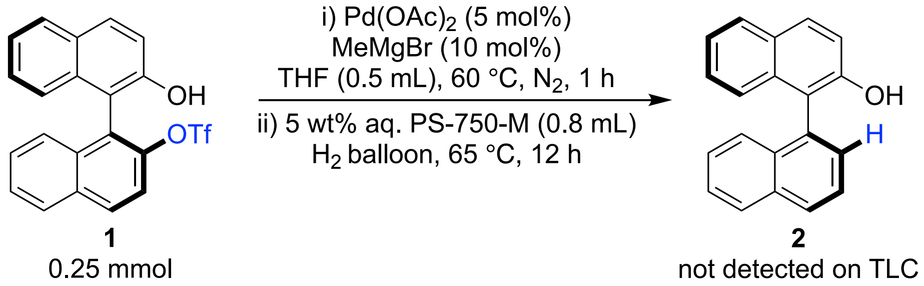

Scheme S1. Regeneration of PdHx study.

7.2. Catalytic base-free detriflation using PdH_x synthesized from K_2PdCl_4

Procedure. $PdH_{0.43}$ nanoparticles were synthesized according to the reported literature[2] using K_2PdCl_4. The synthesized nanoparticles were tested for catalytic base-free triflation using the procedure described in Section 8.1 (Scheme S2)

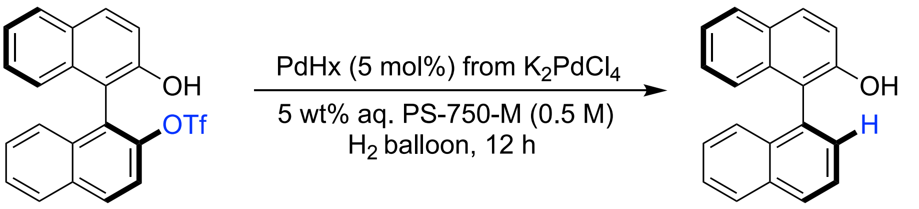

1
0.25 mmol

2
not detected on TLC

Scheme S2. Base-free detriflation with PdH_x synthesized from K_2PdCl_4.

8. BASIC NATURE OF $PdH_{0.43}$: CATALYTIC ACTIVITY

8.1. Catalytic base-free detriflation

Procedure. To an oven-dried vial (4.0 mL) containing a PTFE-coated magnetic stir bar, $Pd(OAc)_2$ (2.8 mg, 5 mol%) was charged. The reaction vial was closed with a subaseal septum. The reaction vial was gently evacuated and backfilled with argon. This cycle was repeated twice more. Dry THF (0.2 mL) was added, and the mixture was stirred at 45 °C for 15 minutes. The mixture was allowed to cool to room temperature, and MeMgBr (9 µL, 0.025 mmol, 3.0 M solution in THF) was added. The mixture turned black at this stage and was stirred at room temperature for an additional 15 minutes. Then, 0.8 mL 5 wt% aq. PS-750-M was introduced. The mixture was allowed to stir for the next 5 min at rt, followed by the addition of (*S*)-2'-hydroxy-[1,1'-binaphthalen]-2-yl trifluoromethanesulfonate) **1** (0.25 mmol), and the septum was wrapped with PTFE tape. A balloon filled with H_2 gas was introduced, and the mixture was stirred at 65 °C for 10 h (Scheme S3). After complete consumption of **1,** as monitored by TLC, the reaction vial was cooled to rt. The septum was removed, and 2 mL EtOAc was added to the reaction mixture to extract the product. The organic layer was separated using a pipette. This extraction was repeated an additional two times. The combined organic layers were dried over Na_2SO_4. Volatiles were evaporated under reduced pressure to obtain the crude product, which was then purified by column chromatography over silica gel using (4:1) hexanes/ethyl acetate as eluent.

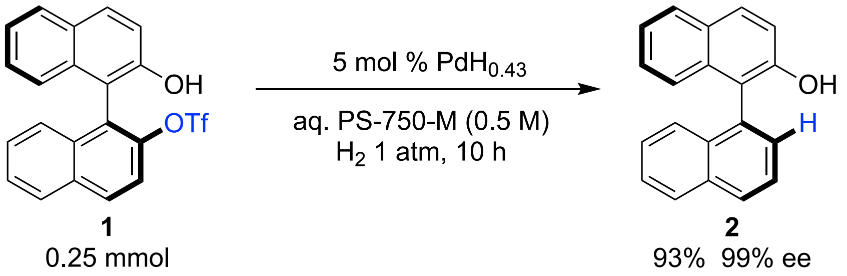

Conditions. 1 (0.25 mmol), $Pd(OAc)_2$ (2.8 mg, 5 mol%), MeMgBr (9 µL, 0.02 mmol, 3.0 M solution in THF), THF (0.2 mL), 5 wt% aq. PS-750-M, 65 °C, H_2 balloon. Reported yield is isolated.

Scheme S3. De-triflation of **1.**

Analytical data of 2 (S)-(1,1'-binaphthalen)-2-ol[3]

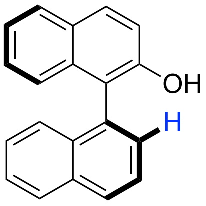

2

White solid, (89%), R_f 0.4 (1:3, ethyl acetate/hexanes) **^{1}H NMR (400 MHz, CDCl$_3$)** δ 8.17 (dd, J = 19.6, 8.3 Hz, 2H), 8.11 – 7.99 (m, 2H), 7.83 (dd, J = 8.3, 7.0 Hz, 1H), 7.75 – 7.66 (m, 2H), 7.59 – 7.47 (m, 4H), 7.44 – 7.39 (m, 1H), 7.27 (d, J = 8.4 Hz, 1H), 5.09 (s, 1H).

^{1}H NMR spectra of 2

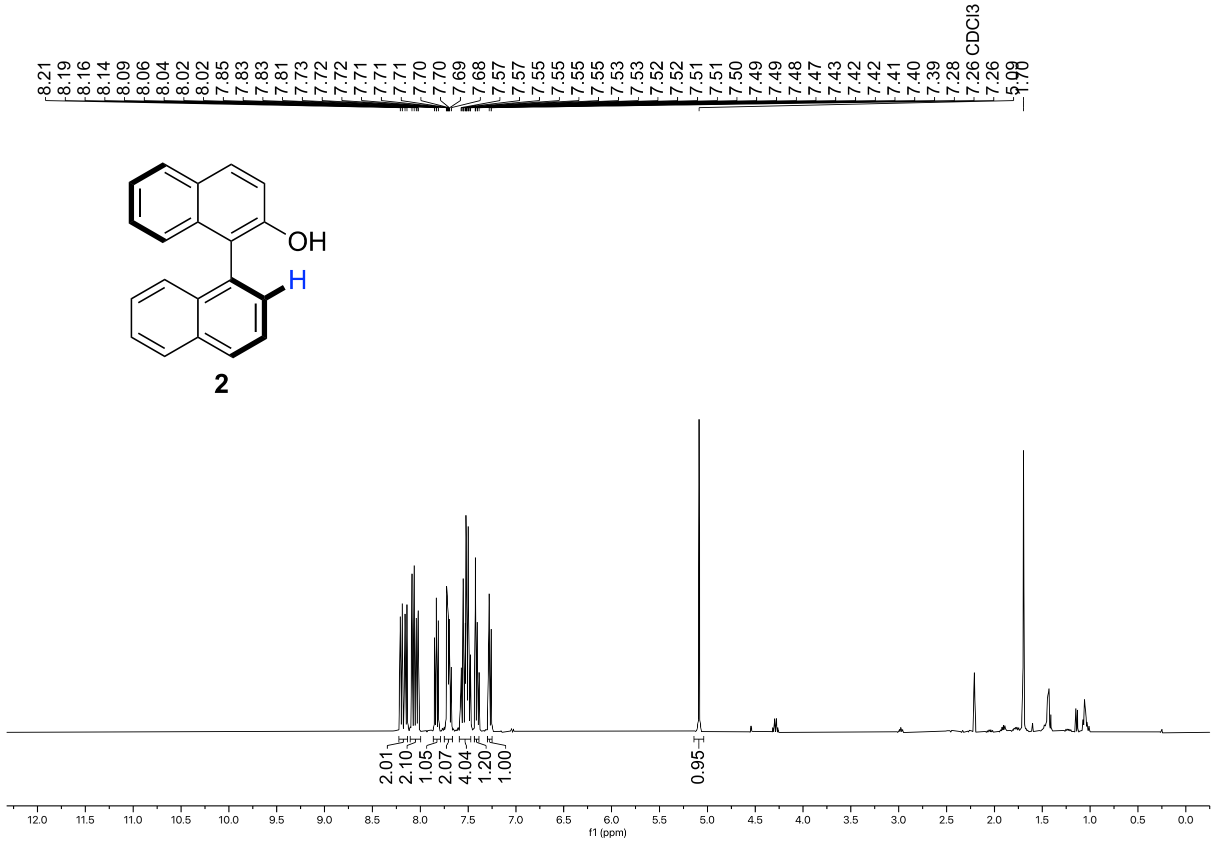

^{13}C NMR spectra of 2

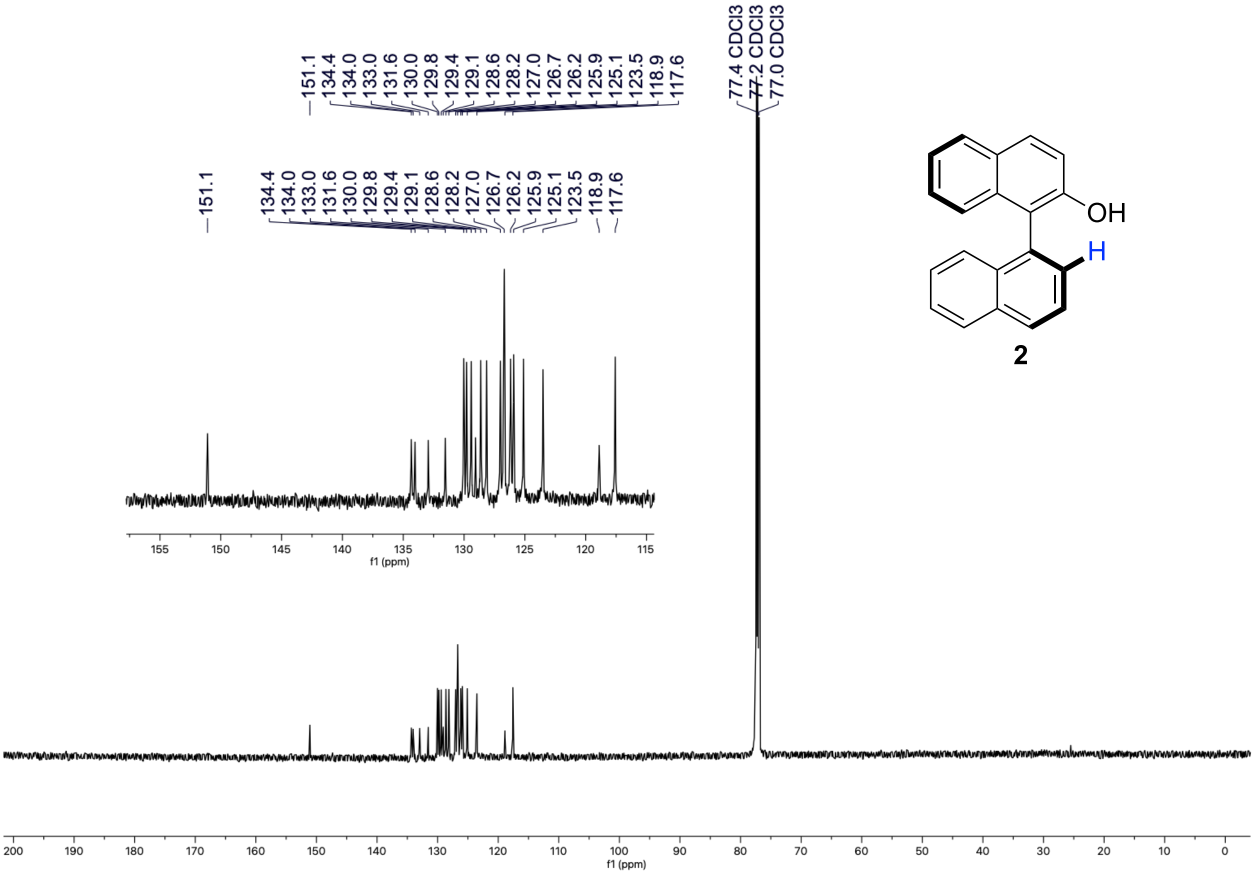

HPLC chromatogram of 1

Racemic 1

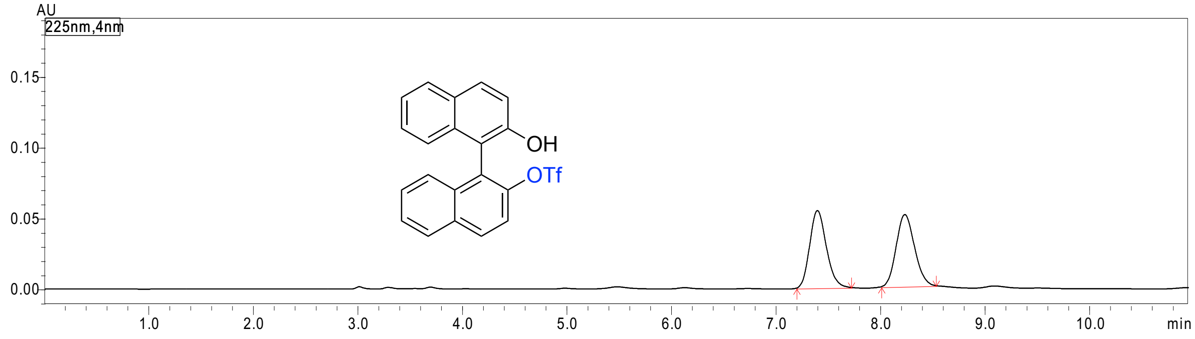

**Peak#**	**Ret. Time**	**Area**	**Height**	**Area%**
1	7.402	604608	54747	49.619
2	8.239	613881	50642	50.381
Total		1218488	105389	100.000

Enantiopure 1 used for catalytic detriflation to obtain 2

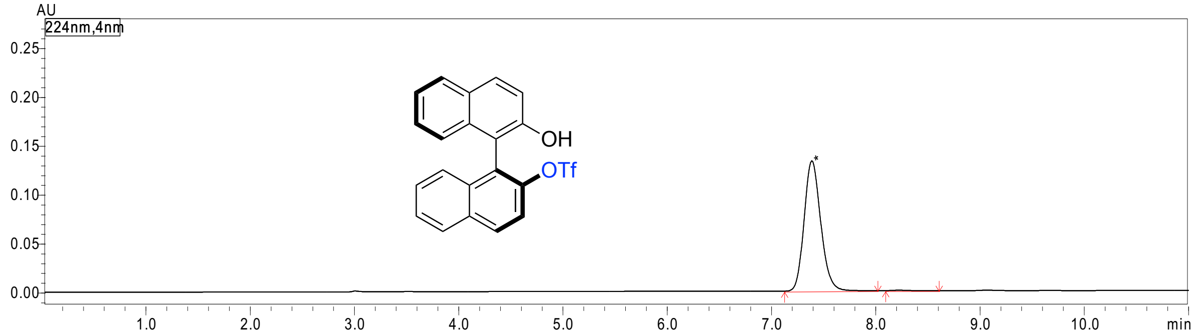

Peak#	Ret. Time	Area	Height	Area%
1	7.394	1473861	133111	99.686
2	8.232	4642	437	0.314
Total		1478503	133548	100.000

HPLC conditions. CHIRALPAK IA column, isopropanol: hexane (1:9), flow rate: 1 mL/min, at rt.

HPLC chromatogram of 2

Racemic 2

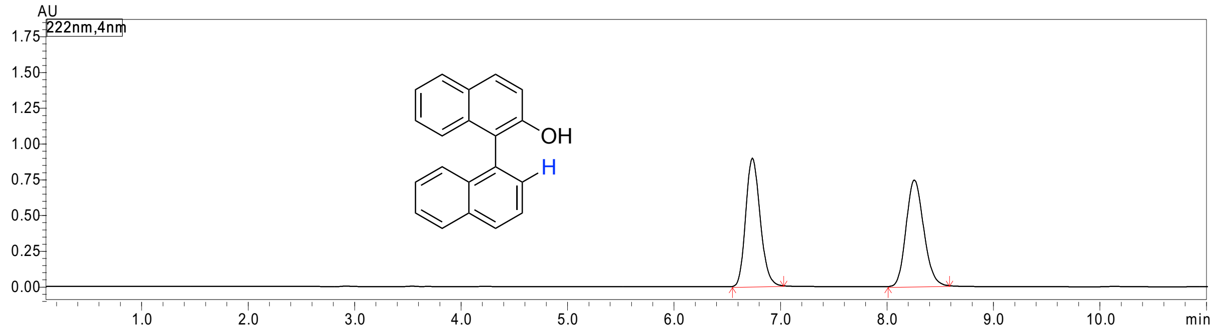

Peak#	Ret. Time	Area	Height	Area%
1	6.742	8423035	896090	49.703
2	8.263	8523747	741868	50.297
Total		16946782	1637958	100.000

Enantiopure 2 obtained from a catalytic reaction of 1 with PdHx

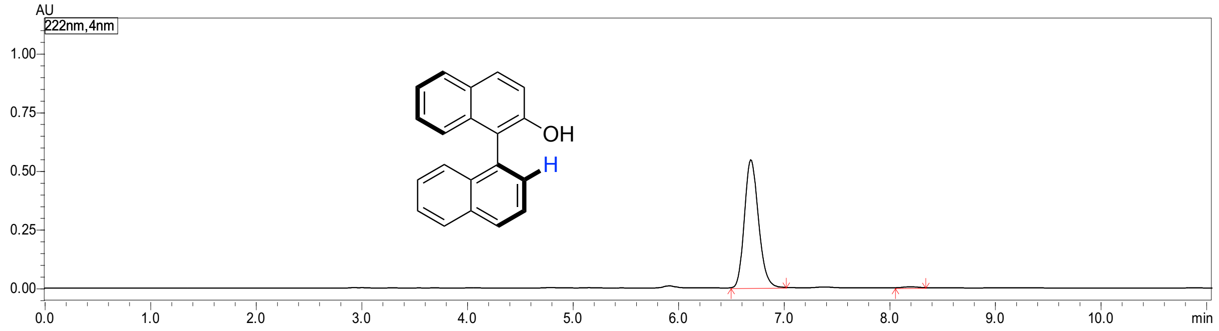

Peak#	Ret. Time	Area	Height	Area%
1	6.691	5046522	545996	99.290
2	8.203	36062	3911	0.710
Total		5082584	549907	100.000

HPLC conditions. CHIRALPAK IA column, isopropanol: hexane (1:9), flow rate: 1 mL/min, at rt.

8.2. Catalytic base-free hydro-debromination

Procedure. To an oven-dried vial (4.0 mL) containing a PTFE-coated magnetic stir bar, $Pd(OAc)_2$ (2.8 mg, 5 mol%) was charged. The reaction vial was closed with a subaseal septum. The reaction vial was gently evacuated and backfilled with nitrogen. This cycle was repeated twice more. Dry THF (0.2 mL) was added, and the mixture was stirred at 45 ^{o}C for 15 minutes. The mixture was allowed to cool to room temperature, and MeMgBr (9 µL, 0.025 mmol, 3.0 M solution in Et_2O) was added. The mixture turned black at this stage and was stirred at room temperature for an additional 15 minutes. Then, 0.8 mL freshly degassed 5 wt% aq. PS-750-M was introduced. The mixture was allowed to stir for the next 5 min at rt, followed by the addition of 4-(4-bromophenyl)morpholine **3** (0.25 mmol), and the septum was wrapped with PTFE tape. A balloon filled with H_2 gas was introduced, and the mixture was stirred at 65 °C for 24 h (Scheme S4). After complete consumption of **3**, as monitored by TLC, the reaction vial was cooled to rt. The septum was removed, and 2 mL EtOAc was added to the reaction mixture to extract the product. The organic layer was separated using a pipette. This extraction was repeated an additional two times. The combined organic layers were dried over Na_2SO_4. Volatiles were evaporated under reduced

pressure to obtain the crude product, which was then purified by column chromatography over silica gel using (5:1) hexanes/ethyl acetate as eluent.

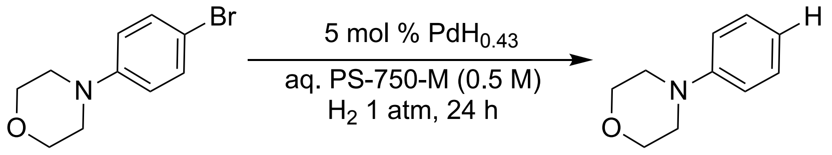

3
0.25 mmol

4
complete conversion on GC-MS
63% isolated yield*

Conditions. 3 (0.25 mmol), $Pd(OAc)_2$ (2.8 mg, 5 mol%), MeMgBr (9 µL, 0.02 mmol, 3.0 M solution in Et_2O), THF (0.2 mL), 5 wt% aq. PS-750-M, 65 °C, H_2 balloon. Reported yield is isolated. *Lower yield due to the volatility of the product.

Scheme S4. Hydro-debromination of **3.**

Analytical data of 4-phenylmorpholine (4) [4]

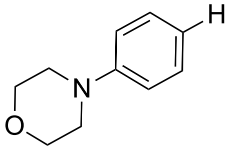

4

White solid, (26 mg, 63%), R$_f$ 0.6 (1:10, ethyl acetate/hexanes) **^{1}H NMR (500 MHz, CDCl$_3$)** δ 7.32 – 7.29 (m, 2H), 6.99 – 6.94 (m, 3H), 3.89 (s, 4H), 3.19 (s, 4H).

^{1}H NMR spectra of 4

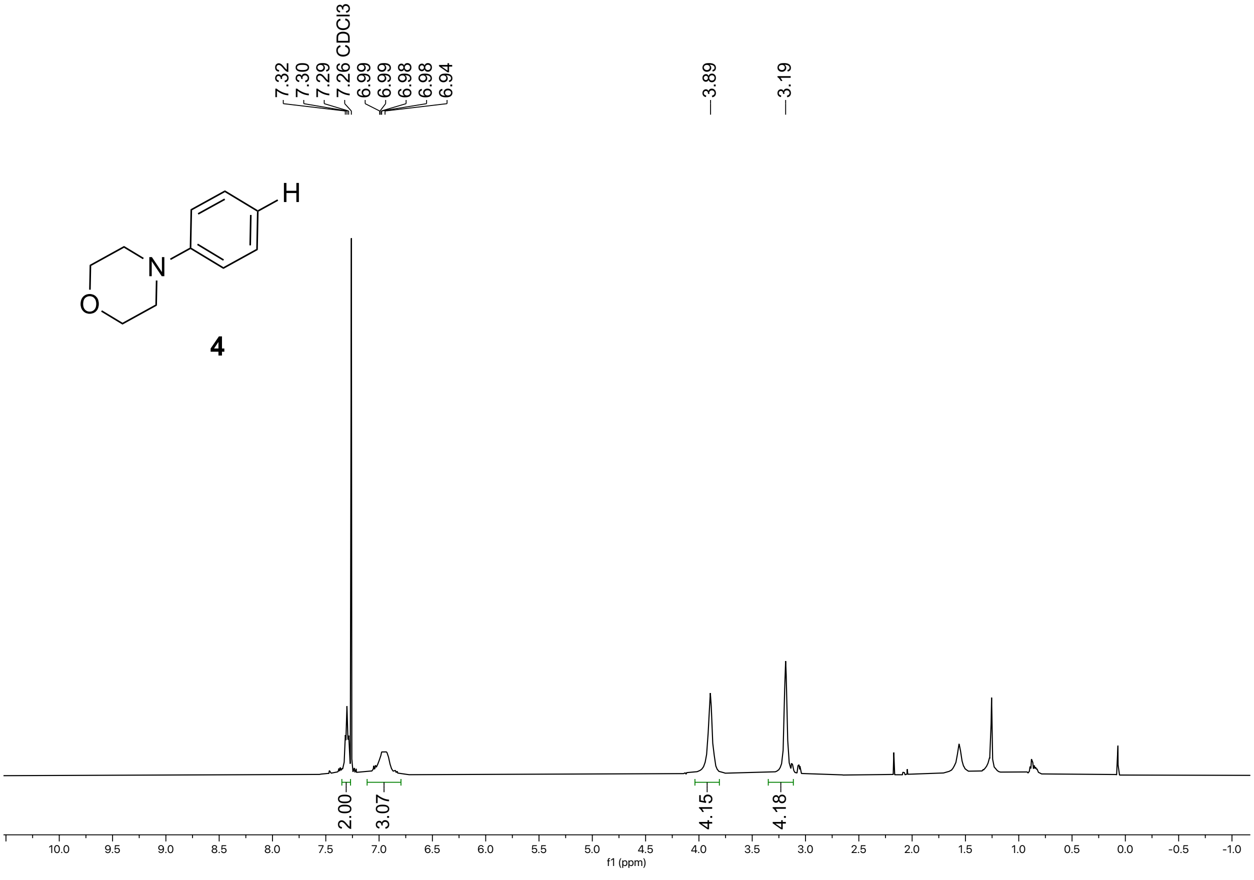

GC-MS chromatogram of 4

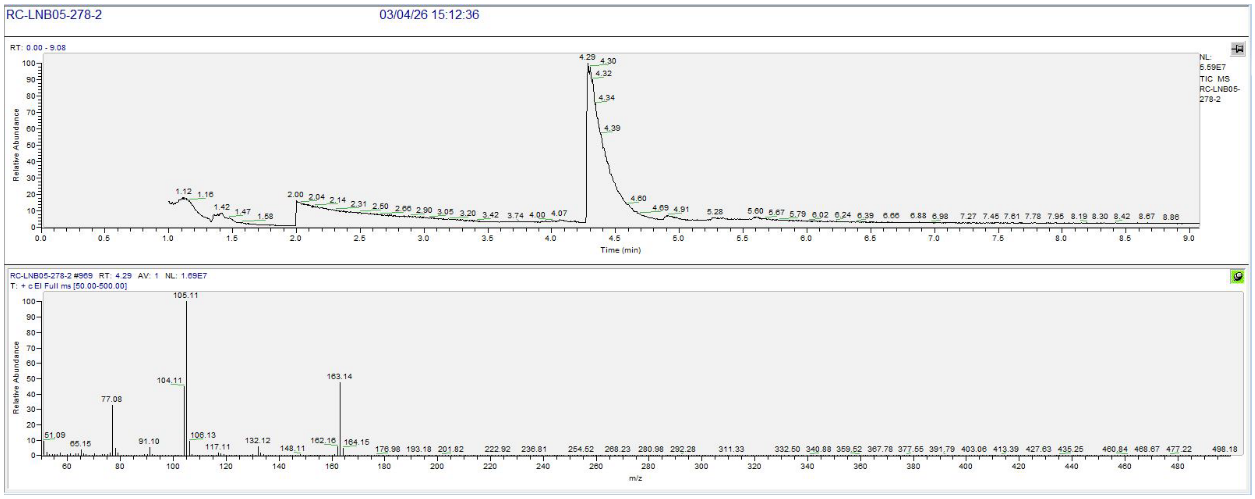

9. REFERENCES

(1) Fulmer, G. R.; Miller, A. J. M.; Sherden, N. H.; Gottlieb, H. E.; Nudelman, A.; Stoltz, B. M.; Bercaw, J. E.; Goldberg, K. I. NMR Chemical Shifts of Trace Impurities: Common Laboratory Solvents, Organics, and Gases in Deuterated Solvents Relevant to the Organometallic Chemist. *Organometallics* **2010**, *29*, 2176–2179 https://doi.org/10.1021/om100106e.

(2) Zhao, Z.; Huang, X.; Li, M.; Wang, G.; Lee, C.; Zhu, E.; Duan, X.; Huang, Y. Synthesis of Stable Shape-Controlled Catalytically Active β-Palladium Hydride. *J. Am. Chem. Soc.* **2015**, *137*, 15672–15675. https://doi.org/10.1021/jacs.5b11543.

(3) Zhang, K.; Oestreich, M. Atroposelective Synthesis of Azobenzenes by Palladium-Catalyzed Cross-Coupling of Racemic Biaryl Triflates and Diazenyl Pronucleophiles. *J. Am. Chem. Soc.* **2025**, *147*, 32329–32334. https://doi.org/10.1021/jacs.5c09097.

(4) Zhang, R.; Hai, J.; Chen, H.; Zhu, M.; Zhang, F. The Position-Tuned Nitrogen Atom in Ni(II)-Metalated Covalent Organic Frameworks Enables Highly Efficient and Sustainable C–N Coupling. *Green Chem.* **2025**, *27*, 13311–13322. https://doi.org/10.1039/D5GC03438H.
